# *Cis*- and *Trans*-variations of Stearoyl-CoA Desaturase Provide New Insights into the Mechanisms of Diverged Pattern of Phenotypic Plasticity for Temperature Adaptation in Two Congeneric Oyster Species

**DOI:** 10.1093/molbev/msad015

**Published:** 2023-01-20

**Authors:** Chaogang Wang, Ao Li, Rihao Cong, Haigang Qi, Wei Wang, Guofan Zhang, Li Li

**Affiliations:** CAS and Shandong Province Key Laboratory of Experimental Marine Biology, Center for Ocean Mega-Science, Institute of Oceanology, Chinese Academy of Sciences, Qingdao, China; Laboratory for Marine Biology and Biotechnology, Pilot National Laboratory for Marine Science and Technology, Qingdao, China; University of Chinese Academy of Sciences, Beijing, China; CAS and Shandong Province Key Laboratory of Experimental Marine Biology, Center for Ocean Mega-Science, Institute of Oceanology, Chinese Academy of Sciences, Qingdao, China; Laboratory for Marine Fisheries Science and Food Production Processes, Pilot National Laboratory for Marine Science and Technology, Qingdao, China; National and Local Joint Engineering Laboratory of Ecological Mariculture, Institute of Oceanology, Chinese Academy of Sciences, Qingdao, China; CAS and Shandong Province Key Laboratory of Experimental Marine Biology, Center for Ocean Mega-Science, Institute of Oceanology, Chinese Academy of Sciences, Qingdao, China; Laboratory for Marine Biology and Biotechnology, Pilot National Laboratory for Marine Science and Technology, Qingdao, China; National and Local Joint Engineering Laboratory of Ecological Mariculture, Institute of Oceanology, Chinese Academy of Sciences, Qingdao, China; CAS and Shandong Province Key Laboratory of Experimental Marine Biology, Center for Ocean Mega-Science, Institute of Oceanology, Chinese Academy of Sciences, Qingdao, China; Laboratory for Marine Biology and Biotechnology, Pilot National Laboratory for Marine Science and Technology, Qingdao, China; National and Local Joint Engineering Laboratory of Ecological Mariculture, Institute of Oceanology, Chinese Academy of Sciences, Qingdao, China; CAS and Shandong Province Key Laboratory of Experimental Marine Biology, Center for Ocean Mega-Science, Institute of Oceanology, Chinese Academy of Sciences, Qingdao, China; Laboratory for Marine Biology and Biotechnology, Pilot National Laboratory for Marine Science and Technology, Qingdao, China; National and Local Joint Engineering Laboratory of Ecological Mariculture, Institute of Oceanology, Chinese Academy of Sciences, Qingdao, China; CAS and Shandong Province Key Laboratory of Experimental Marine Biology, Center for Ocean Mega-Science, Institute of Oceanology, Chinese Academy of Sciences, Qingdao, China; University of Chinese Academy of Sciences, Beijing, China; Laboratory for Marine Fisheries Science and Food Production Processes, Pilot National Laboratory for Marine Science and Technology, Qingdao, China; National and Local Joint Engineering Laboratory of Ecological Mariculture, Institute of Oceanology, Chinese Academy of Sciences, Qingdao, China; CAS and Shandong Province Key Laboratory of Experimental Marine Biology, Center for Ocean Mega-Science, Institute of Oceanology, Chinese Academy of Sciences, Qingdao, China; Laboratory for Marine Biology and Biotechnology, Pilot National Laboratory for Marine Science and Technology, Qingdao, China; University of Chinese Academy of Sciences, Beijing, China; National and Local Joint Engineering Laboratory of Ecological Mariculture, Institute of Oceanology, Chinese Academy of Sciences, Qingdao, China

**Keywords:** phenotypic plasticity, temperature adaptation, oleic acid content, stearoyl-CoA desaturase, *cis*- and *trans*-regulation, oysters

## Abstract

The evolution of phenotypic plasticity plays an essential role in adaptive responses to climate change; however, its regulatory mechanisms in marine organisms which exhibit high phenotypic plasticity still remain poorly understood. The temperature-responsive trait oleic acid content and its major gene stearoyl-CoA desaturase (*Scd*) expression have diverged in two allopatric congeneric oyster species, cold-adapted *Crassostrea gigas* and warm-adapted *Crassostrea angulata*. In this study, genetic and molecular methods were used to characterize fatty acid desaturation and membrane fluidity regulated by oyster *Scd*. Sixteen causative single-nucleotide polymorphisms (SNPs) were identified in the promoter/*cis*-region of the *Scd* between wild *C. gigas* and *C. angulata*. Further functional experiments showed that an SNP (g.-333C [*C. gigas* allele] >T [*C. angulata* allele]) may influence *Scd* transcription by creating/disrupting the binding motif of the positive *trans*-factor Y-box factor in *C. gigas*/*C. angulata*, which mediates the higher/lower constitutive expression of *Scd* in *C. gigas*/*C. angulata*. Additionally, the positive *trans*-factor sterol-regulatory element–binding proteins (*Srebp*) were identified to specifically bind to the promoter of *Scd* in both species, and were downregulated during cold stress in *C. gigas* compared to upregulated in *C. angulata*. This partly explains the relatively lower environmental sensitivity (plasticity) of *Scd* in *C. gigas*. This study serves as an experimental case to reveal that both *cis*- and *trans*-variations shape the diverged pattern of phenotypic plasticity, which provides new insights into the formation of adaptive traits and the prediction of the adaptive potential of marine organisms to future climate change.

## Introduction

Phenotypic plasticity, the expression of different phenotypes from the same genotype in response to environmental variation, as well as genetic variations are both crucial for increasing the fitness of organism under changing environmental conditions (Hereford 2009; Pfennig et al. 2010; Markov and Ivnitsky 2016; Kelly 2019). Many studies have suggested that phenotypic plasticity can act as an evolutionary force to foster new traits and species, and may even fuel adaptive radiation (West-Eberhard 1989; Pigliucci and Murren 2003; Pfennig et al. 2010; Moczek et al. 2011). Different genotypes typically demonstrate different environmentally contingent phenotypic responses (Sultan and Bazzaz 1993; Kingsolver et al. 2004), which provide heritable variations on which selection can act to promote an evolutionary change in phenotypic plasticity (Ehrenreich and Pfennig 2016). Therefore, changes in phenotypic plasticity are an essential component of the evolutionary responses to climate change, which implies that the genetic variations underlying environment-induced phenotypic plasticity could be an important predictor of the vulnerabilities of species and populations to climate-driven decline or extinction (Rockman and Kruglyak 2006; Kelly 2019). However, the elucidation of regulating mechanisms associated with divergence of plasticity is still in infancy for marine organisms.

Adaptive phenotypic plasticity is achieved through various mechanisms that involve almost all physiological levels and systems, including morphology, behavior, physiology, and development (Berthold et al. 1992; Weinig et al. 2003; Gutteling, Doroszuk, et al. 2007; Gutteling, Riksen, et al. 2007; Doroszuk et al. 2009; Rodriguez et al. 2012; Van Dijk and Hautekèete 2014). Phenotypic plasticity is frequently accompanied by changes in gene expression (Aubin-Horth and Renn 2009). Owing to the development of high-throughput techniques, gene expression has been widely used as a mini-phenotype and proxy for study in phenotypic plasticity (Bedulina et al. 2013; Grishkevich and Yanai 2013; Kenkel et al. 2013; Dayan et al. 2015). The *cis*- and *trans*-variations that alter gene regulation are important contributors to the evolution of gene expression (Pfennig and Ehrenreich 2014), which abundantly exists in diverged populations (Bell et al. 2013; Osada et al. 2017; Verta and Jones 2019), subspecies (Davidson and Balakrishnan 2016; Zhong et al. 2019; Campbell et al. 2020), and related species (Tirosh et al. 2006). The integration of comparative genomic and transcriptomic data has revealed that under selection, the genotypic variation disproportionately correlates with environmental responsiveness and occurs in the promoter sequence of plastic genes (Tirosh et al. 2006; Tirosh and Barkai 2008; Grishkevich et al. 2012). *Cis*-regulatory variants may create or disrupt *trans*-factor-binding motif, altering the environmental sensitivity (plasticity) of gene expression (Wittkopp and Kalay 2012). In addition, *trans*-factor variations are involved in the molecular regulation of highly plastic and complex gene expression changes, such as functional mutation or expression changes, in facing environmental disturbance (Gibson and Weir 2005; Ray et al. 2011; Gehan et al. 2015; Monroe et al. 2016; Lamrabet et al. 2019; Sabino-Pinto et al. 2019; Xiao et al. 2019). Although many theoretical and laboratory studies have observed diverged patterns of phenotypic plasticity in marine organisms (Losos et al. 2000; Pfennig and Murphy 2000; de Jong 2005; Wund et al. 2008; Scoville and Pfrender 2010; Robinson 2013; Laland et al. 2014; Schlichting and Wund 2014), the underlying genetic and molecular mechanisms, including identification of regulatory elements and regulatory network of the environmentally responsive genes are still preliminary.

The environmentally responsive trait with a known major gene provides an ideal system to reveal the mechanisms of diverged phenotypic plasticity. In response to temperature variations, which are a major environmental factor reflecting climate change, animals regulate the fatty acid composition (unsaturated fatty acids [UFAs]/saturated fatty acids [SFAs] ratio) of cell membranes to adjust fluidity and phase to maintain key biophysical properties (Tiku et al. 1996; Mendoza 2014; Ma, Li, et al. 2015; Winnikoff et al. 2021). Stearoyl-CoA desaturase (SCD), as rate-limiting enzyme of the biosynthesis of monounsaturated fatty acids, introduces the first double bond at the Δ9 position of palmitoyl-CoA (16:0) and stearoyl-CoA (18:0), which are then converted to palmitoleoyl-CoA (16:1) and oleoyl-CoA (18:1), respectively (Ntambi 1995; Tocher et al. 1998; Cohen et al. 2002; Lee et al. 2004; Nakamura and Nara 2004). The expression of the *Scd* gene is strongly correlated with low temperatures, playing a significant role in enhancing the adaptative capability of organisms, including nematode (Svensk et al. 2013), amphibia (Al-attar et al. 2019), and fish (Hsieh et al. 2003; Hsieh and Kuo 2005; Ma, Qiang, et al. 2015; Xu et al. 2015), under cold stress. *Scd* gene expression was tightly regulated by sterol-regulatory element–binding proteins (SREBPs), peroxisome proliferator-activated receptors (PPARs) and liver × receptors in vertebrates (Sampath and Ntambi 2005; Lengi and Corl 2012; Ma and Corl 2012; Shi, Luo, Yao, et al. 2013). At present, most studies about *Scd* in marine organisms focused on the function identification in the fatty acid desaturation, its regulatory variations mediated the environment-induced expression plasticity still remain unclear.

The related species that have adapted to different environments can be used to refine trait expression and uncover regulatory variations to understand the role of the evolution of plasticity in the evolutionary molecular mechanisms (Kenkel et al. 2013; Dayan et al. 2015; Ghaffari et al. 2019). Oyster, as key species in marine ecosystem and global aquaculture, has evolved high phenotypic plasticity to adapt to the heterogeneous intertidal zone (Guofan et al. 2012; Li et al. 2021; Wang, Li, Wang, Zhang, et al. 2021). *Crassostrea gigas* and *Crassostrea angulata* are two allopatric congeneric oyster species, which have adapted to relatively cold and warm habitats (Northern and Southern coast of China; Haiyan et al. 2008; Ren et al. 2010; Wang et al. 2010). These two species serve as an important model for investigating the evolution of temperature adaption (Sebens 2002; Li et al. 2017, 2021). *Crassostrea gigas* and *C. angulata* adapt to an environmental temperature gradient, reflected by differential thermal tolerance and differential fatty acid content and composition (especially oleic acid C18:1; Ghaffari et al. 2019; Wang, Li, Wang, Cong, et al. 2021). And previous study has observed the adaptive divergence of plasticity in environmentally responsive genes between *C. gigas* and *C. angulata* (Li et al. 2019, 2021). Additionally, the integration of resequencing data and transcriptome analysis of *C. gigas* and *C. angulata* revealed that the environmentally responsive gene *Scd*, showed a strong selective sweep in the upstream noncoding region of *C. angulata* (Li et al. 2021). Therefore, *Scd* and fatty acid composition act as temperature responsive and highly plastic gene and trait and can be quantified to investigate the genetic basis and molecular mechanisms of divergence of phenotypic plasticity.

In this study, we applied genetic and molecular methods to characterize the role of *Scd* in fatty acid desaturation and membrane fluidity, screen and validate that the causative *cis*-mutations regulated differentially constitutive expression, and identify that the *trans*-factor regulated differential phenotypic plasticity in response to low temperature between *C. gigas* and *C. angulata*. This study serves as an experimental case study to reveal the genetic basis and molecular mechanisms underlying a diverged pattern of the phenotypic plasticity, which provides new insights into the significance of plasticity in the formation of adaptive traits and the prediction of the adaptive potential for marine organisms in facing to rapid climate change.

## Results

### Functional Characterization of Oyster *Scd*

**Fig. 1.**
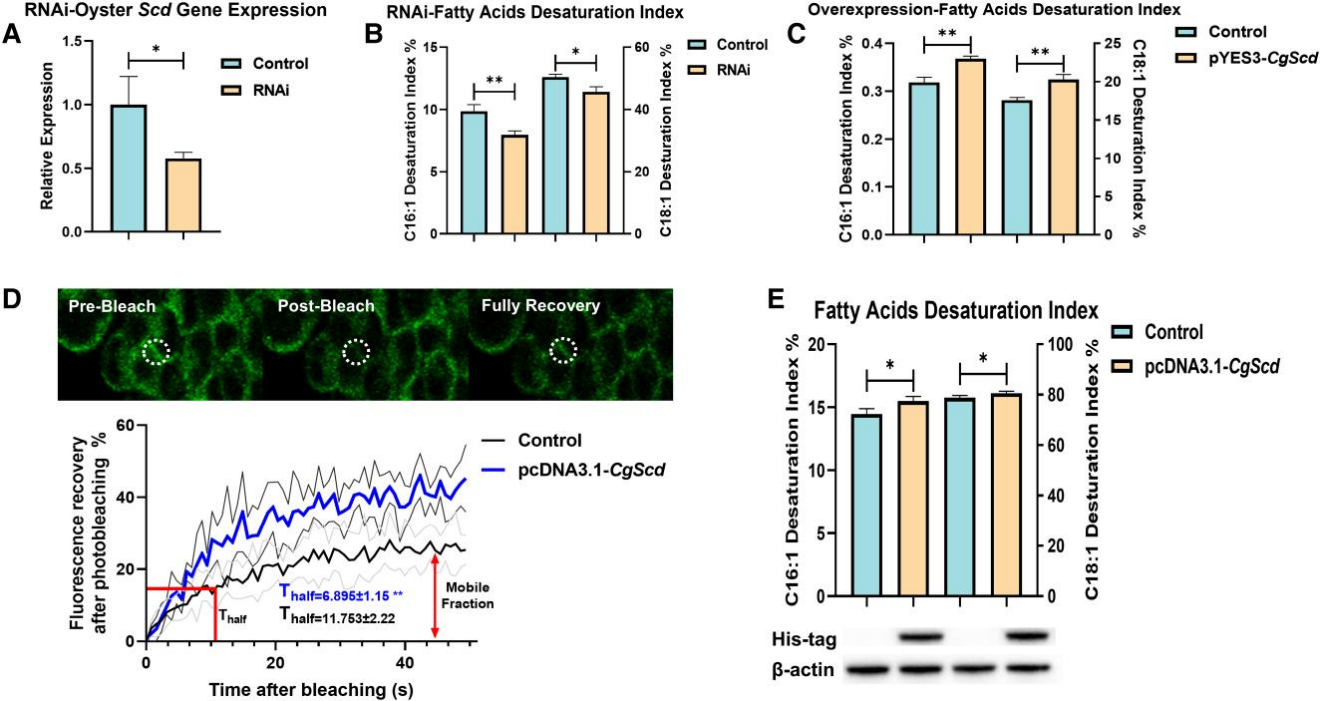
The characterization of the function of the oyster stearoyl-CoA desaturase (*Scd*) gene. (*A*) The relative gene expression of oyster *Scd* in gill tissues after three repeated injections (72 h) of siRNA-321 in RNA interference experiments (*n* = 3). RNAi and control in the legend represent the oyster was injected with siRNA and nonsense strands, respectively. The error bars represent the standard error mean (SEM). (*B*) The measurements of fatty acids desaturation indexes in gill tissues in RNAi experiments (*n* = 3). The left vertical axis represents the palmitoleic acid (C16:1) desaturation index, and the right vertical axis represents the oleic acid (C18:1) desaturation index. RNAi and control in the legend represents the groups were injected with siRNA and nonsense strands, respectively. The error bars represent the SEM. (*C*) The measurements of fatty acid desaturation indexes in the oyster *Scd* overexpression experiment in yeast cells after supplemented with a mixture of stearic acid and palmitic acid (*n* = 3). The pYES3-CgScd and control in the legend represent that the yeast cells were introduced with yeast overexpression plasmid pYES3 ligated with CDS of oyster *Scd* and empty pYES3 plasmid, respectively. The left vertical axis represents the palmitoleic acid (C16:1) desaturation index, and the right vertical axis represents the oleic acid (C18:1) desaturation index. The error bars represent the SEM. (*D*) The results of the FRAP experiment (*n* = 5). The position marked by the white dotted circle is the bleached region at pre-bleaching, post-bleaching, and full-recovery time points during the experiment. The fitted curves show the average fluorescence over time, and error bars represent the SEM. The blue line represents the treatment group, which was transfected with the pcDNA3.1-*CgScd* plasmid, and the black line represents the control group, which was transfected with the empty pcDNA3.1 plasmid. Mobile fraction in the legend represents the maximum recovery. And *T*_half_ in the legend represents the average time point that fluorescence recovers to half of the maximum recovery. (*E*) The fatty acid desaturation indexes in FRAP experiment. The pcDNA3.1-*CgScd* and control in the legend represent the groups were transected with the overexpression plasmid pcDNA3.1 ligated with CDS of oyster *Scd* and empty pcDNA3.1 plasmid. The left vertical axis represents the palmitoleic acid (C16:1) desaturation index, and the right vertical axis represents the oleic acid (C18:1) desaturation index. *CgScd* protein expression was confirmed by western blotting. β-Actin served as a loading control. Significant differences among groups were marked with *P < 0.05 and **P < 0.01. “ns” indicates non-significant differences.

To comprehensively characterize the function of oyster *Scd*, we conducted the RNA interference (RNAi), overexpression in yeast cells and fluorescence recovery after photobleaching (FRAP) experiments. The pilot experiment on oyster *Scd* RNAi experiment showed that the siRNA “*Scd*-321” significantly interfered with *CgScd* expression after 72 h (supplementary fig. S1, Supplementary Material online). Therefore, *Scd*-321 and 72 h were considered the most effective siRNA and adequate time for RNA interference, respectively. The formal experiment demonstrated that *Scd* expression was significantly downregulated after three duplicate injections of *Scd*-321 compared with that in the control group (fig. 1*A*, *P* < 0.05); thus, the palmitoleic acid (C16:1) desaturation and oleic acid (C18:1) desaturation indexes also decreased (fig. 1*B*, supplementary table S5, Supplementary Material online; *P* < 0.05). Moreover, the C16:1 and C18:1 desaturation indexes were significantly higher in yeast cells overexpressing *CgScd* than in control cells (fig. 1*C*, supplementary table S6, Supplementary Material online; *P* < 0.01). And the figure 1*D* clearly demonstrated that the process of cell membrane being bleached by strong laser and then recovering in the FRAP experiment. The mobile fraction and *T*_half_ were used to measure the percentage of the movable part of the membrane in the overall membrane and the recovery rate of cell membrane after bleaching. It was showed that the mobile fraction and *T*_half_ values were significantly higher and faster, respectively, in the pcDNA3.1-*CgScd* group than in the control group (fig. 1*D*, *P* < 0.01). The corresponding fatty acid measurements also demonstrated that the C16:1 and C18:1 desaturation indexes were significantly higher in the *CgScd* overexpression group than in the control group (fig. 1*E*, supplementary table S7, Supplementary Material online; *P* < 0.05), and protein expression was confirmed using western blotting (fig. 1*E*). Taken together, the results proved that oyster *Scd* gene catalyzes C16:1 and C18:1 synthesis, and then regulates cell membrane fluidity.

### Screening for Potential Causative Genetic Variations

**Fig. 2.**
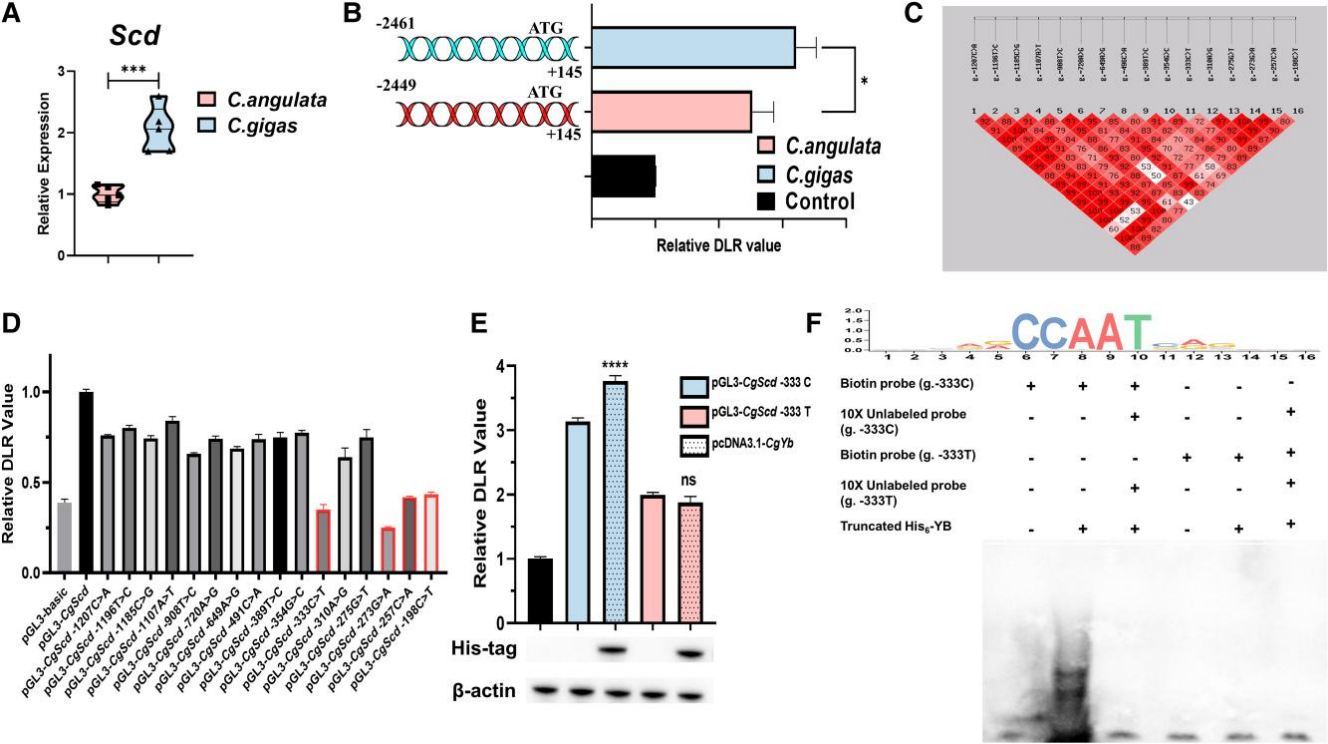
The screening and functional characterization of *cis*-variations in *Scd* for the two oyster species. (*A*) The relative expression of *Scd* in gill tissues of *C. gigas* and *C. angulata* in a wild environment after one-generation common garden experiment in Northern coast of China (37°39′N, Muping, Shandong province; *n* = 5). The error bars represent the SEM. (*B*) The relative dual-luciferase reporter (DLR) values of cells transfected with the promoter of the *Scd* gene (*n* = 3). The pGL3-*CgScd* and pGL3-*CaScd* in the legend represent that the pGL3-basic plasmid ligated with about 2.5*k* promoter sequence of *Scd* from *C. gigas* and *C. angulata*. And the control represents the control group transfected with empty pGL3-basic plasmid. The error bars represent the SEM. (*C*) The results of linkage disequilibrium block analysis based on the genotyping data (Sanger sequencing of 16 SNPs) from *C. gigas* and *C. angulata*. The number on the block represents *D*′ value. (*D*) The relative DLR values of cells transfected with mutated plasmid in the single-site mutation experiments (*n* = 3). The labels of *x*-axis represent 16 SNPs mutation (from *C. gigas* allele to *C. angulata* allele) plasmid based on pGL3-*CgScd*. Red border color marks four SNPs with the strongest mutational effects. The error bars represent the SEM. (*E*) The relative DLR value of cells co-transfected with pcDNA3.1-*CgYb*, pGL3-*CgScd* (g.-333C, *C. gigas* allele) and pGL3-*CgScd* (g.-333T, *C. angulata* allele) (*n* = 3). The error bars represent the S.E.M. *CgYb* protein expression was confirmed by western blotting. β-Actin served as a loading control. (*F*) Electrophoretic mobility shift assay of the *CgScd* promoter with His_6_-*Cg*YB. The g.-333C probe and g.-333T represent the biotinylated and unlabeled sequences carrying g.-333C (*C. gigas* allele)>T (*C. angulata* allele) SNP site. The unlabeled probes added at 10-fold excess were used to verify specific DNA–protein interactions (lines 3, 6). The upper panel represents the binding sites logo of the Y-box factor, which was obtained from the JASPAR database and redrew by TBtools. Significant differences among groups were marked with *P < 0.05, ***P < 0.001, and ****P < 0.0001. “ns” indicates non-significant differences.

We compared the constitutive expression of *Scd* between the two oyster species and found that *Scd* expression in *C. gigas* was significantly higher than that in *C. angulata* (fig. 2*A*, *P* < 0.001). Therefore, we amplified the promoter of *Scd* in *C. gigas* (2,461 bp) and *C. angulata* (2,449 bp) and constructed into the pGL3-basic vector. Based on the luciferase reporter assay, the *Scd* promoter of *C. gigas* had higher transcriptional activity than that of *C. angulata* (fig. 2*B*, *P* < 0.05). We amplified the promoter sequence of each species and mixed it into a pool separately for high-throughput sequencing to screen single-nucleotide polymorphisms (SNPs) and InDels within the ∼2 kb promoter region of oyster *Scd*. Quality analysis showed that library construction and sequencing results were reliable (supplementary table S8, Supplementary Material online). A total of 17 SNPs and 1 InDel were identified between the 2 species (*P* < 0.05; table 1). Subsequently, more samples (50 wild *C. gigas* and 50 wild *C. angulata* collected from their natural habitats) were used for genotyping by Sanger sequencing to further validate the significance of the difference in the above variation sites. Our results revealed 16 sites in the *Scd* promoter with significantly differential allele frequency between the 2 species (table 2; *P* < 0.05, *χ*^2^ test). Additionally, strong linkage disequilibrium was detected among the 16 variation sites (fig. 2*C*).

**Table 1.** Statistics of Genotype Frequency by Mixed-Pool Target Amplicon Sequencing.

Name	POS	REF	ALT	Ca		Cg		*P*-value
				REF	ALT	REF	ALT	
**Site1**	g.-1107	A	T	4	1,471	296	1,356	0
**Site2**	g.-908	T	C	3	1,791	284	1,555	0
**Site3**	g.-649	A	G	2	1,451	280	1,814	0
**Site4**	g.-198	C	T	0	2,023	259	2,265	0
**Site5**	g.-273	G	A	0	2,136	222	2,190	1.02637197267061e-318
**Site6**	g.-1185	C	G	1	1,118	299	1,278	1.68414160202775e-311
**Site7**	g.-1196	T	C	1	1,042	299	1,267	1.9016E-303
**Site8**	g.-720	A	G	0	1,447	245	1,743	4.4679E-303
**Site9**	g.-1207	C	A	1	975	301	1,256	3.7053E-297
**Site10**	g.-310	A	G	1	2,256	153	2,153	1.5219E-244
**Site11**	g.-333	C	T	1	2,231	107	2,130	5.0721E-186
**Site12**	g.-1464	T	A	0	1,420	135	999	5.6949E-183
**Site13**	g.-491	C	A	356	1,198	258	1,972	2.7313E-141
**Site14**	g.-257	C	A	614	1,362	253	2,206	8.1955E-116
**Site15**	g.-275	G	T	674	1,475	219	2,188	1.1852E-101
**Site16**	g.-1381	ATATAACTG	A	7	2,123	54	1,082	1.64292E-87
**Site17**	g.-354	G	C	655	1,519	65	2,109	1.22071E-33
**Site18**	g.-389	T	C	513	1,307	61	0	3.52413E-33

Note.—Ca, *Crassostrea angulata*; Cg, *Crassostrea gigas*.

**Table 2.** Statistics of Genotype Frequency by the Sanger Sequencing.

		REF	ALT	Cg			Ca			*χ* ^2^-value	*P*-value
				0/0	0/1	1/1	0/0	0/1	1/1		
**Site1**	g.-1107	A	T	41	0	9	2	0	48	62.056305	1.78E-15
**Site2**	g.-908	T	C	35	2	13	0	5	49	53.940887	2.23E-12
**Site3**	g.-649	A	G	36	1	13	0	7	42	55.786499	9.08E-13
**Site4**	g.-198	C	T	47	1	0	13	24	13	54.422119	1.05E-11
**Site5**	g.-273	G	A	15	8	27	0	1	48	26.317028	2.00E-06
**Site6**	g.-1185	C	G	17	6	27	1	1	48	23.673653	7.44E-06
**Site7**	g.-1196	T	C	13	8	29	0	1	48	23.125015	9.77E-06
**Site8**	g.-720	A	G	36	1	13	4	12	33	43.597694	3.75E-10
**Site9**	g.-1207	C	A	12	7	31	0	1	49	20.549999	3.52E-05
**Site10**	g.-310	A	G	50	0	0	10	13	27	66.666664	0.00E + 00
**Site11**	g.-333	C	T	36	1	13	4	8	38	43.299347	4.35E-10
**Site12**	g.-1464	T	A	3	0	47	1	0	49	1.041667	0.307484
**Site13**	g.-491	C	A	35	1	14	3	5	41	42.862854	5.40E-10
**Site14**	g.-257	C	A	50	0	0	38	9	3	13.636364	0.001104
**Site15**	g.-275	G	T	50	0	0	11	15	24	63.934425	2.45E-14
**Site16**	g.-1381	ATATAACTG	A	2	0	48	0	0	50	2.040816	0.15319
**Site17**	g.-354	G	C	36	1	13	2	8	40	49.620213	1.90E-11
**Site18**	g.-389	T	C	34	2	14	3	8	38	40.643944	1.62E-09

Note.—Ca, *Crassostrea angulata*; Cg, *Crassostrea gigas*. Red font represents sites with significant differences in genotype frequency after verification by Sanger sequencing.

### Functional Analysis of the Detected Genetic Variations Reveals a Causative SNP

To determine the effect of the 16 identified variation sites effect in the oyster *Scd* promoter, we mutated each site in the original plasmid (pGL3-*CgScd*) and performed a luciferase reporter assay. Each mutation (*C. gigas* allele to *C. angulata* allele) resulted in a significant decrease in the transcriptional activity compared with the unmutated plasmid (pGL3-*CgScd*, fig. 2*D*). Four SNPs (g.-333C>T, g.-273G>A, g.-257C>A, and g.-198C>T) with the most potent effects were selected for subsequent DNA pull-down experiments. The quality of nuclear protein extracts was verified by western blotting using a nuclear protein marker (histone H3). The band of the nuclear protein extracts was clear, whereas those of the cytoplasmic protein extracts band showed weak color (supplementary fig. S5, Supplementary Material online). The bound proteins were observed by silver staining after polyacrylamide gel electrophoresis. Silver staining showed that most of the binding proteins were consistently isolated, which may be due to the low concentration of binding proteins (supplementary fig. S6, Supplementary Material online). Therefore, we detected all proteins in the entire lane based on mass spectrometry (MS) and found that the g.-333C, g.-333T, g.-273G, g.-273A, g.-257C, g.-257A, g.-198C, and g.-198T and empty magnetic bead groups pulled 67, 32, 52, 8, 27, 37, 16, 24, and 41 proteins, respectively (supplementary fig. S7, Supplementary Material online). After excluding the binding proteins pulled by empty magnetic bead, the common binding proteins pulled by the wild type and the mutant probe and their respective specific binding proteins were identified as potential binding proteins. We screened the candidate transcriptional factors from the identified binding proteins pulled commonly and specifically by each wild-type probe and mutant probe based on the functional annotation of oyster genome. The results showed that the Y-box factor was among the binding proteins specifically pulled by g.-333C probe (supplementary table S9, Supplementary Material online).

Then, we conducted luciferase reporter assay and electrophoretic mobility shift assay (EMSA) experiment to identify the regulatory relationship and binding specificity between the Y-box factor and the promoter of *Scd* in *C. gigas*. Upon *Cg*YB overexpression, the relative luciferase activity of pGL3-*CgScd* (g.-333C, *C. gigas* allele) significantly increased (fig. 2*E*, *P* < 0.0001). However, there was no significant variation difference in luciferase activity when *Cg*YB was co-transfected with pGL3-*CgScd* (g.-333T, *C. angulata* allele; fig. 2*E*). Protein overexpression was confirmed by western blotting (fig. 2*E*). The EMSA demonstrated that the addition of truncated His_6_-*Cg*YB (supplementary fig. S8, Supplementary Material online) delayed the electrophoretic mobility of the probe containing the *C. gigas* allele region (g.-333C); however, it did not interact with the *C. angulata* allele region (g.-333T) probe (fig. 2*F*, lanes 2 and 5). Moreover, the unlabeled probe competition assays out-competed the specific interactions, thus eliminating the presence of shifted bands and indicating a specific interaction between truncated His_6_-*Cg*YB and the *C. gigas Scd* promoter region (fig. 2*F*, lane 3).

### The Expression Plasticity and Regulation of *Scd* During Short-Term Cold Stress

**Fig. 3.**
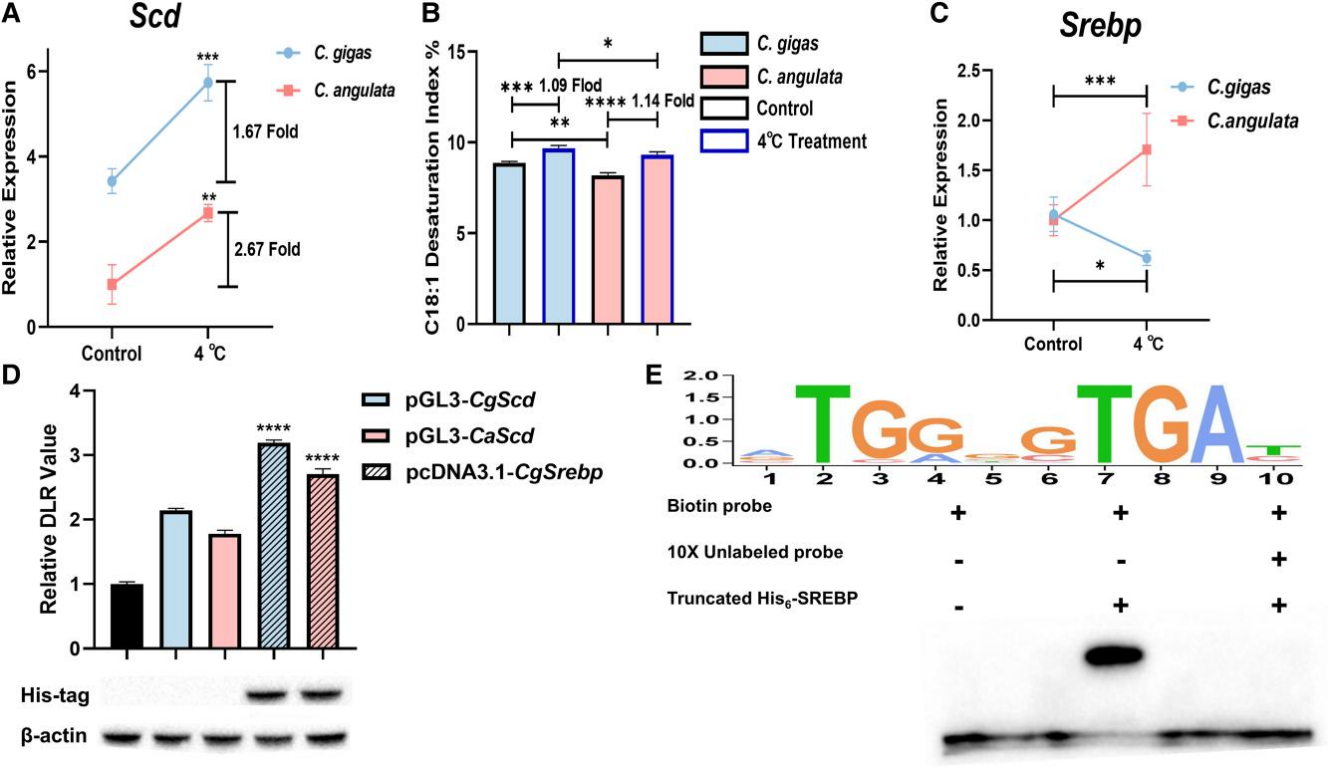
The differential expression of the *trans*-factor *Srebp* regulates *Scd* plasticity during short-term cold stress in *C. gigas* and *C. angulata*. (*A*) The relative expression of *Scd* during short-term cold stress in gill tissues of *C. gigas* and *C. angulata* (*n* = 3). Blue border represents the cold stress treatment. The error bars represent the SEM. (*B*) The oleic acid (C18:1) desaturation index in *C. gigas* and *C. angulata* during short-term cold stress (*n* = 3). The error bars represent the SEM. (*C*) The relative expression of *Srebp* during short-term cold stress in *C. gigas* and *C. angulata* (*n* = 3). The error bars represent the SEM. (*D*) The relative DLR values of cells co-transfected with pcDNA3.1-*CgSrebp*, pGL3-*CgScd* and pGL3-*CaScd* (*n* = 3). The error bars represent the SEM. *CgSrebp* protein expression was confirmed by western blotting. β-Actin served as a loading control. (*E*) Electrophoretic mobility shift assay of the *CgScd* promoter with His_6_-*Cg*SREBP. The unlabeled probes added at 10-fold excess were used to verify specific DNA–protein interactions (line 3). The upper panel represents the binding sites logo of SREBP, which was obtained from the JASPAR database and redrew by TBtools. Significant differences among groups were marked with *P < 0.05, **P < 0.01, ***P < 0.001 and ****P<0.0001. “ns” indicates non-significant differences.

A short-term cold stress experiment was conducted to observe the plasticity of *Scd* in two species, and the results revealed that the expression of *Scd* was significantly higher in the group under cold stress conditions than in the control group in both species (fig. 3*A*, *P* < 0.01), indicated that the *Scd* gene was significantly induced by low temperature (*P* < 0.0001). The expression of *Scd* in *C. gigas* was consistently significantly higher than that in *C. angulata* (*P* < 0.01); however, the upregulation magnitude of *Scd* in *C. angulata* (2.67-fold) was greater than that in *C. gigas* under cold stress conditions (1.67-fold; fig. 3*A*). Additionally, the C18:1 desaturation index was consistent with the gene expression (fig. 3*B*, supplementary table S10, Supplementary Material online); it increased under cold stress, and was significantly higher in *C. gigas* than in *C. angulata* under both normal (control groups; *P* < 0.01) and cold stress conditions (*P* < 0.05). Additionally, the magnitude of the upregulation of *C. angulata* (1.14-fold) was slightly greater than that of *C. gigas* (1.09-fold; fig. 3*B*). Although the C16:1 desaturation index showed an upward trend after cold stress, there was no significant difference between *C. gigas* and *C. angulata* in the control groups and those under cold stress conditions (supplementary fig. S9 and table S10, Supplementary Material online), which may be due to the high preference for stearic acid in *Scd* (Gonzalez-Rovira et al. 2022). To identify the causative regulatory factors that influence *Scd* differential plasticity expression, we performed quantitative real-time polymerase chain reaction (qRT-PCR) experiments to measure and compare oyster *Ppar* and *Srebp* gene expression between two species under cold stress conditions. The results showed that oyster *Ppar* gene expression was not induced by low temperature; however, it was consistently significantly more expressed in *C. gigas* than in *C. angulata* (supplementary fig. S10, Supplementary Material online; *P* < 0.0001). The expression of the oyster *Srebp* gene was not significantly different between two species under normal conditions. However, under cold stress, the expression of *Srebp* gene was significantly increased in *C. angulata* (*P* < 0.001, fig. 3*C*) but significantly downregulated in *C. gigas* (*P* < 0.05, fig. 3*C*), showing an opposite regulatory trend in its expression. In addition, the luciferase reporter assay showed that the relative luciferase activity driven by the *CgScd* and *CaScd* promoter was significantly increased by SREBP expression (*P* < 0.001, fig. 3*D*). The EMSA experiment demonstrated that the addition of truncated His_6_-CgSREBP (supplementary fig. S11, Supplementary Material online) delayed the electrophoretic mobility of the oyster *Scd* promoter region (fig. 3*E*, lane 2). The unlabeled probe assay out-competed the specific interactions, thereby eliminating the presence of the shifted band (fig. 3*E*, lane 3).

## Discussions

### The Diverged Pattern of Plasticity for Low-Temperature Adaptation is Represented by Difference of Oleic Acid Content and *Scd* Expression Between the Two Oyster Species

In this study, gene function and membrane fluidity experiments provided direct evidence that *Scd* is involved in the biosynthesis of UFAs and temperature adaptation in oyster, which is consistent with the studies in model species (Cohen et al. 2002; Lee et al. 2004; Shi, Li, et al. 2013; Igal and Sinner 2021; Tuthill et al. 2021), fish (Hsieh et al. 2004) and copepods (Jung et al. 2016). Subsequently, short-term cold stress experiments demonstrated that the oleic acid (C18:1) desaturation index was induced by low temperatures in both species, and *C. gigas* had a significantly higher constitutive content with a slightly lower magnitude of upregulation compared with *C. angulata*. This phenomenon of fatty acid composition adjustment of cell membranes (increasing the ratio of the UFAs/SFAs of cell membrane) at low temperatures, termed homeoviscous adaptation, has been widely recognized in bacteria, archaea, and eukaryotes (Sinensky 1974; Cossins and Prosser 1978; Anderson et al. 1981; Shmeeda et al. 2002; Miyazaki and Ntambi 2003; Holthuis and Menon 2014; Mendoza 2014). Moreover, the expression of the metabolic gene *Scd* expression further demonstrated that it was also induced by low temperatures in both species, and *C. gigas* had significantly higher expression of *Scd* than *C. angulata* under both normal temperature (control) and cold conditions; however, it exhibited a lower magnitude of upregulation under cold stress compared with that in *C. angulata* (*C. gigas* 1.67-fold; *C. angulata* 2.67-fold). The diverged trait means and plasticity of oleic acid (C18:1) content and *Scd* gene expression in two oyster species support our previous findings that fatty acid composition/*Scd* is an important temperature adaptive trait/gene and that adaptive divergence of plasticity occurs (Li et al. 2021; Wang, Li, Wang, Cong, et al. 2021). The content of UFAs and their corresponding metabolic gene *Scd* constitute an ideal system to study the underlying molecular mechanisms and genetic basis of diverged pattern of plasticity for temperature adaptation.

The trade-offs between trait mean (constitutive expression of *Scd*/content of C18:1 in this study) and plasticity (plastic expression of *Scd*/content of C18:1 in this study) between *C. gigas* and *C. angulta* is consistent with the phenomenon of negative relationship between trait mean and plasticity (van Heerwaarden and Kellermann 2020), demonstrating a common diverged pattern of phenotypic plasticity which has been widely observed in various environment-induced traits between populations and relative species, including morphology (Aubret and Shine 2009; Gunter et al. 2017; Albarran-Melzer et al. 2020), physiology (Tomanek and Somero 2000; Hua et al. 2015; Phillips et al. 2016; Comte and Olden 2017; Kellermann et al. 2017), behavior (Shaw et al. 2007; Foster 2013), and gene expression (Scoville and Pfrender 2010; Barshis Daniel et al. 2013; Bedulina et al. 2013; Kenkel and Matz 2017; Levis et al. 2017; Li et al. 2019; Yang et al. 2021). The higher constitutive expression and lower plastic expression of *Scd* in *C. gigas* may ultimately lead to genetic assimilation in which an environment-triggered adaptive trait becomes “fixed” or is constitutively expressed in a population or species (Waddington 1953; Wittkopp et al. 2004; Yeh and Price 2004; Pigliucci et al. 2006; Crispo 2007; Wund 2012; Bedulina et al. 2013; Kelly 2019). However, owing to the long divergence time of 2.7 Ma (Ren et al. 2010) between *C. gigas* and *C. angulata*, changes in plasticity with regards to their ancestor remains unknown, and evolution mode of the plasticity (genetic assimilation) in these two species cannot be concluded solely based on the trade-offs between the constitutive expression and plastic expression (environmental sensitivity) of *Scd*, which may be confirmed by multiple generations of reciprocal transplantation experiments in future. In summary, the comparison of the regulatory mechanism of *Scd*, the key gene of the focal trait oleic acid, between the two congeneric oyster species that served as experimental case, could shed light on the genetic and molecular mechanisms of evolution of phenotypic plasticity for temperature adaptation.

### *Cis*-Regulatory Element Variations Mediate the Change in the Constitutive Expression of *Scd*

In previous study, we found that the noncoding regions showed higher sequence divergence in selected environmental plastic genes between *C. gigas* and *C. angulata* (Li et al. 2021). Therefore, we further identified and characterized the regulatory elements, focusing on the promoter architecture of *Scd* in the two species. We found a significantly higher transcriptional activity of the promoter of *C. gigas* than that of *C. angulata*. Sixteen causative mutations with strong linkage disequilibrium located within the promoter of *Scd* were screened, and they directly increased the transcriptional activity. This suggests that the *cis*-variations in the promoter were fixed to regulate the expression of *Scd* for low-temperature adaptation, thereby leading to higher constitutive expression of *Scd* in *C. gigas*. Dense variations in *cis*-regulatory regions account for the dominant part of constitutive gene expression across different species and populations (Wittkopp et al. 2004, 2008; Grishkevich et al. 2012; Tangwancharoen et al. 2018), which may be explained by more pronounced pleiotropy reduction compared with that mediated by coding mutations for selection (Wray 2007). Therefore, serval omics and resequencing studies have identified the cis-region with numerous genetic variations as the selection hot spot (Chesler et al. 2005; Cheung et al. 2005; Hubner et al. 2005; DeCook et al. 2006; Wang et al. 2006). In this study, we identified genetic variations in *cis*-regulatory elements and functionally characterized the causative mutation. The Y-box factor, which is a nucleic acid–binding protein with an evolutionary conserved cold shock domain, specifically binds to the g. −333 C site (*C. gigas* allele) and positively regulates the transcriptional activity of the *Scd* promoter but does not interact with g.-333T (*C. angulata* allele). The Y-box factor positively activates *Scd* transcription and specifically binds to “CCAAT” *cis*-element in human cultured keratinocytes (Didier et al. 1988; Zhang et al. 2001; Eliseeva et al. 2011). And our previous transcriptome had demonstrated that the Y-box factor was not induced by environmental stress and there was no difference between *C. gigas* and *C. angulata* (Li et al. 2021; Wang, Li, Wang, Cong, et al. 2021). Therefore, our findings suggest that the mutation of binding sites for a constitutively active transcription factor (Y-box factor in this study) might lead to increased redundancy in gene expression under different environmental cues, ultimately resulting in the decoupling of genes expression from the environment (Pfennig and Ehrenreich 2014). We propose that *cis*-regulatory variations may regulate the gene expression by creating or disrupting transcriptional binding sites, then involves in the changes of constitutive expression of plasticity pattern.

### *Trans*-Factor Variations Mediates Change in the Environmental Sensitivity (Plasticity)

The starting point for studying the mechanisms underlying genetic assimilation is understanding the molecular causes of phenotypic plasticity (Ehrenreich and Pfennig 2016). Phenotypic plasticity is frequently accompanied by changes in gene expression (Aubin-Horth and Renn 2009) and involved in the binding of transcriptional factors to gene promoter or other regulatory elements (Gibson and Weir 2005; Ray et al. 2011; Lamrabet et al. 2019; Sabino-Pinto et al. 2019; Xiao et al. 2019). In this study, we revealed that the SREBP *trans*-factor was positively regulated and specifically bound to *Scd* in both two oyster species. This is consistent with previous studies showing that the *trans*-factor SREBP can activate genes related to fatty acid synthesis such as *Scd* (Tabor et al. 1998; Shimano 2001). It is also regulated by temperature stress to maintain energy homeostasis and membrane fluidity in human HepG2 cells (Shechter et al. 2003), fission yeast (*Saccharomyces pombe*) (Robichon and Dugail 2007), mice (Adlanmerini et al. 2019), and freshwater fish *Onychostoma macrolepis* (Deng et al. 2019). However, *Srebp* showed an opposite expression trend where it was upregulated in *C. angulata* but downregulated in *C. gigas* during short-term cold stress experiments. This downregulation partly explains the lower of environmental sensitivity (plasticity) of *Scd* in *C. gigas* under cold stress. Therefore, this study is the first to report that *Srebp* responds to low temperatures and regulates *Scd* expression in marine organisms. This case study supports that environment-induced *trans*-factors, such as *Srebp*, are important sensors and amplifiers whose changes in expression during ambient condition variations directly impact the divergence of plasticity of downstream genes, such as *Scd*, to environment changes (Ohama et al. 2017).

Another potential transcriptional factor, *Ppar*, did not respond to low temperature; however, its expression was consistently significantly higher in *C. gigas* than in *C. angulata* under both control and cold stress conditions. *Ppar*, a member of the nuclear receptor superfamily activated by ligands, positively regulates *Scd* expression in mice (Yao-Borengasser et al. 2008; O'Neill et al. 2020), goat (Shi, Luo, Zhu, et al. 2013), human HepG2 cells (Saliani et al. 2013), and bovines (Zhang et al. 2020). Therefore, we speculate that its stable high expression in *C. gigas* may be related to the higher constitutive expression of *Scd* compared with that in *C. angulata*.

This study support that the complex hierarchical regulatory network composed of *trans*-factors, as sensory system and transduction pathways, plays a central role in the divergence of plasticity, which provides great selection targets to alter their expression, thereby changing plastic and constitutive expression of downstream genes (Gibson and Weir 2005; Ray et al. 2011; Liu et al. 2018; Lamrabet et al. 2019; Li and Howell 2021; Kidokoro et al. 2022). However, the molecular mechanisms and genetic basis for the differential expression of *trans*-factors (*Srebp* and *Ppar*) in responses to environmental variations need to be further investigated.

## Conclusion

**Fig. 4.**
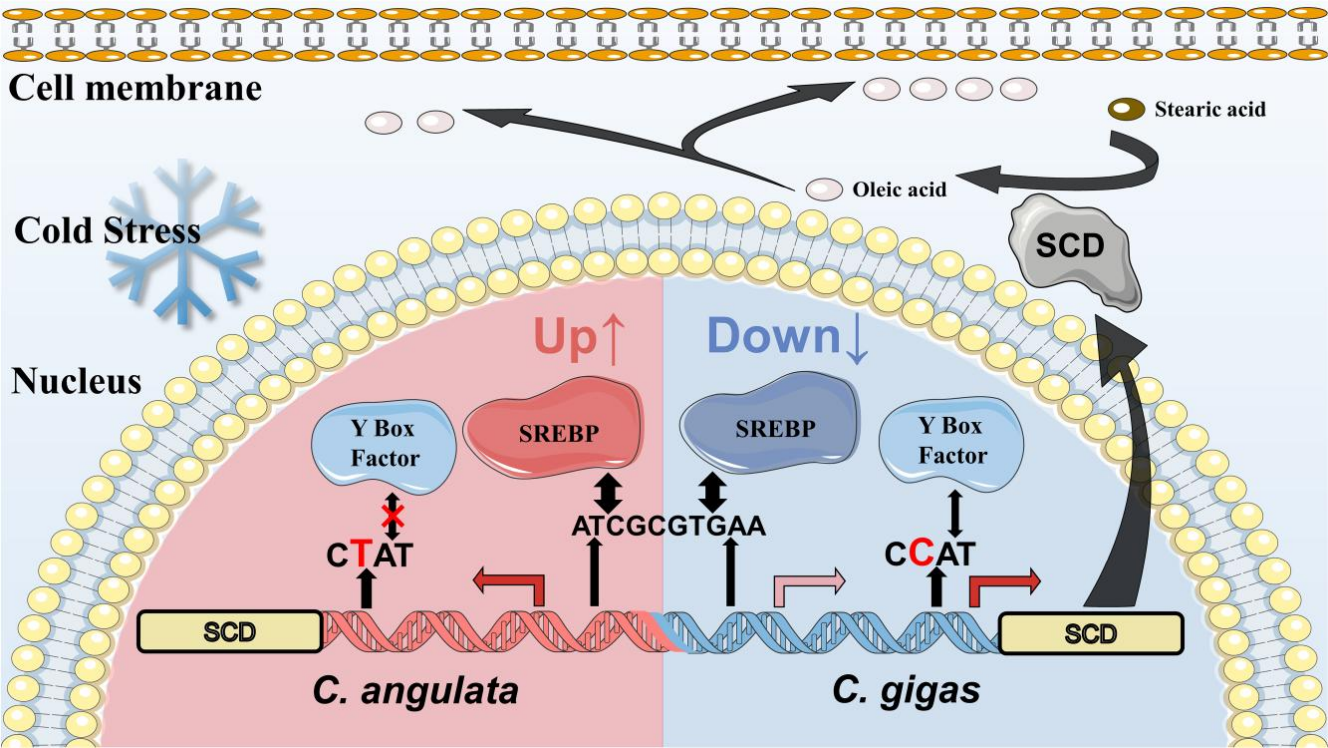
Schematic representation of *Scd* plastic expression pattern in *C. gigas* and *C. angulata* shaped by *cis*-variations and *trans*-factors. The significantly higher constitutive expression of *Scd* and content of oleic acid in relatively cold-adapted *C. gigas* compared with relatively warm-adapted *C. angulata* are regulated by *cis*-variations in the promoter of *Scd*. Among these, the SNP (g.-333C>T) can create/disrupt the regulatory element of the positively *trans*-factor Y-box factor in *C. gigas*/*C. angulata*, resulting in differential transcriptional activity. The cold-induced positively *trans*-factor *Srebp* exhibited an opposite expression trend (increased in *C. angulata* and decreased in *C. gigas*), which may partly mediate the lower expression plasticity of *Scd* in *C. gigas* than *C. angulata* under cold stress. And oyster SCD catalyzes the stearic acid to oleic acid, which involves in the unsaturated fatty acid acyl chain of cell membrane phospholipids, then increases the membrane fluidity to maintain normal biophysical properties of cell membrane in response to low temperature.

In this study, we revealed that *C. gigas* adapts to the relatively stable cold environment (Northern China), exhibits higher *Scd* constitutive expression and its metabolic product oleic acid, as well as lower plasticity to the cold condition compared with the warm-adapted *C. angulata* (Southern of China), trade-offs between trait mean and plasticity demonstrating a common diverged pattern of the phenotypic plasticity shaped by long-term temperature gradient. The causative *cis*- and *trans*-variations mediating the expression of *Scd* were identified as the molecular regulatory mechanisms underlying the divergence of plasticity. A schematic of the molecular mechanisms leading to the diverged plasticity of *Scd* expression in *C. gigas* and *C. angulata* is provided in figure 4. Fixed *cis*-regulatory variants account for the constitutive expression of *Scd* and oleic acid content by changing the binding motif of Y-box factor, whereas the *trans*-factor *Srebp* (induced by low temperature) positively regulates and specifically binds to *Scd*, and its differential expression may partly mediate variations in the plasticity of *Scd*. This study serves as a proxy to reveal the genetic and molecular mechanisms underlying a common divergent pattern of phenotypic plasticity for environmentally responsive trait, which will further deepen the understanding for the evolution of phenotypic plasticity. It will also provide new insights into the formation of adaptive traits and the prediction of the adaptive potential of marine organisms to future climate change.

## Materials and Methods

### Experimental Animals

The design of spawning experiment was based on our previous study (Li et al. 2017). Briefly, two congeneric oyster species (*C. gigas* and *C. angulata*) were collected and sampled (50 *C. gigas* and 50 *C. angulata*) from their natural habitat in Qingdao (35°44′ N) and Xiamen (24°33′ N), respectively. Previous study showed that the higher mean air (8.7 °C) and sea surface temperatures (6.6 °C) at the southern site than those at the northern site over 2 years (Li et al. 2017). The one-generation common garden experiment was conducted to alleviate the environmental effects (Sanford and Kelly 2011). We collected and mixed the eggs of 30 mature females and divided them into 30 beakers for each species. Then, the sperm from each of the 30 mature males were crossed separately with eggs in each beaker. After four months, the F_1_ progenies were transported to the sea of Muping City (37°39′N, Shandong province, China) for grow-out culture. Two months later, we sampled 15 adult progeny oysters and gill tissues of each species in situ for subsequent experiments and collected 30 *C. gigas* for the RNAi experiment and 30 *C. gigas* and *C. angulata* oysters for short-term cold stress experiment. Additionally, the wild oysters used for Sanger sequencing were sampled from the Yellow Sea (35°44′N, Qingdao, Shandong province, China) and the East China Sea (24°33′N, Xiamen, Fujian Province, China; supplementary fig. S1, Supplementary Material online; Liu et al. 2020).

### RNAi Experiment

Small interfering RNA (siRNA) was synthesized by GenePharma (Shanghai, China) and used for the RNAi experiments (sequences of the siRNA are shown in supplementary table S1, Supplementary Material online). Thirty *C. gigas* were cleaned and reared in a 500-l tank for acclimation, and fed with 4 g/m^3^ commercial Spirulina powder once per day (Wang, Li, Wang, Cong, et al. 2021). After 1 week, individuals were anesthetized as previously described (Suquet et al. 2009) (500 g MgCl_2_ + 5 l seawater + 5 l freshwater) and then divided into two groups: the siRNA (*n* = 15) and the negative control (NC) groups (*n* = 15). Then, 100 μl of 10 μg/100 μl siRNA and 10 μg/100 μl NC strands were injected in the muscles of individuals in two groups, respectively. The interference time and selected siRNA were optimized in the pilot experiment (supplementary fig. S2, Supplementary Material online). Based on the results of the pilot experiment, we selected the most effective siRNA “Scd-321” and repeated the injection two times after 72 h in a formal experiment, which is necessary for fatty acid accumulation. After 9 days, gill tissues were collected for gene expression measurement and fatty acid measurements. The qRT-PCR was performed using the ABI 7500 Fast Real-Time PCR System (Applied Biosystems, USA) using the Taq Pro Universal SYBR qPCR Master Mix (Vazyme Biotech, China). The primers used for gene expression detection are listed in supplementary table S2, Supplementary Material online. The desaturation indexes of palmitoleic acid (C16:1) and oleic acid (C18:1) which was also used to demonstrate the enzyme activity of SCD (Chajes et al. 2011) were calculated as follows:

(1) $$C16:1\;\mathrm{or}\;C18:1\;\mathrm{desaturation}\;\mathrm{index}\;=\frac{C16:1\;\mathrm{or}\;C18:1}{C16:1+C18:1+C16:0+C18:0}$$

### Overexpression Experiment

Total RNA was extracted from *C. gigas* gill tissues using TRIzol reagent. Then, the first-strand cDNA was synthesized using a HiScript® III 1st Strand cDNA Synthesis Kit (Vazyme Biotech). PCR fragments corresponding to the open reading frames (ORFs) of *CgScd* were amplified using 2 × Phanta Max Master Mix (Vazyme Biotech) using forward and reverse primers (supplementary table S3, Supplementary Material online) containing restriction sites for *Apa*I (New England Biolabs, USA) which were designed based on the oyster genome (GenBank accession no. GCA_011032805.1) (Qi et al. 2021). The amplified DNA fragments were gel purified and ligated into a similarly restricted pYES3-CT vector to yield the plasmid construct, pYES3-*CgScd*. The ligation products were transformed into DH5α competent cells (Tsingke Biotechnology, China) to screen for the presence of recombinants by DNA sequence (Tsingke Biotechnology). pYES3-*CgScd* was purified from DH5α using the SPARKeasy Endofree Midi Plasmid Kit (Shandong Sparkjade Biotechnology, China). The pYES3 and purified pYES3-*CgScd* vectors were introduced into competent *Saccharomyces cerevisiae* (INVSc1 strain) cells (AngYuBio, China). The transformants were selected on synthetic dropout medium tryptophan and confirmed by DNA sequencing. Yeast transformed with pYES3 (control) and pYES3-*CgScd* vectors was cultured for 24 h in a growth medium (2% glucose; Solarbio, China) at 30 °C. The cultures were centrifuged at 500 × g for 2 min at room temperature and then resuspended in the induction medium (2% galactose; Solarbio), and cultured until an optical density (OD)_600_ of 0.6 was reached. The cultures were supplemented with a mixture of stearic acid and palmitic acid (Sigma-Aldrich, USA). After 2 days, yeast cells were harvested, washed, and freeze-dried for subsequent fatty acid measurements. The fatty acid desaturation index calculation was performed using equation (1).

### FRAP Experiment

Plasmid construction was performed as described above. Briefly, the ORF of *CgScd* was ligated into pcDNA3.1-myc-HisA (supplementary table S3, Supplementary Material online; MiaoLing Plasmid Platform, China). HEK-293 T cells (Procell Life Science & Technology, China) were plated in a Glass Bottom Cell Culture dish (NEST, China) 1 day before transfection and were cultured under adherent conditions in high-glucose DMEM (Biological Industries, Israel) and 10% fetal bovine serum (FBS; Biological Industries). Cells were transfected with 2.5 µg (per well) pcDNA3.1-*CgScd* plasmid using Lipofectamine 3000 (Invitrogen, USA) and then stained with 1 μM 4′-(trimethylammonium) diphenylhexatriene (AAT Bioquest, USA). FRAP experiments were performed using a Zeiss LSM710 laser scanning confocal microscope with an EC Plan-Neofluar 40X objective lens. The HEK293T cells expressing the pcDNA3.1-*Cg*SCD were photobleached over a region of interest (10-pixels radius) using 50 iterations of the 405 nm laser with 100% laser power transmission. Images (digital zoom 6X) were collected using a pixel dwell time of 1.58 μs. The fluorescence recovery and *T*_half_ values of the bleached region were calculated as follows: Firstly, all fluorescence values were adjusted based on the slope of the decreasing fluorescence of reference non-photobleached region to compensate the bleaching caused by repetitive scanning and imaging. Then, the lowest value after bleaching was subtracted from all adjusted fluorescence values, thereby setting the post-bleach fluorescence to zero. And the average value of the five measurements of preceded bleaching and last measurement were set to the pre-bleach value and the maximum recovery which is corresponded to the mobile fraction. The time point that fluorescence recovers to half of the maximum recovery was identified the halftime of recovery (*T*_half_ value). *CgScd* expression was determined using western blotting. HEK293T cells were collected for fatty acid measurement. The fatty acid desaturation index was calculated using equation (1).

### Western Blotting

HEK293T cell transformants were extracted using centrifugal force. The cells were then resuspended in M-PER™ Mammalian Protein Extraction Reagent containing protease inhibitor (Thermo Fisher Scientific, USA) at room temperature for 20 min. After the supernatant protein was collected by centrifugation, 4X protein loading buffer (GenScript Biotech, China) was added and denatured at 100 °C for 10 min. Proteins were resolved by 12% SDS-PAGE and transferred onto 0.45 nm pore polyvinylidene fluoride (PVDF) membrane (Millipore, USA) using an eBlot™ L1 wet transfer (GenScript Biotech). Membranes were blocked and incubated with anti-6x His-tag antibody and secondary antibodies (goat anti-mouse; Abcam, UK) using eZwest Lite Automated Western Device (GenScript Biotech). Membranes were then incubated with ECL western blotting substrate (Solarbio) and captured using the Molecular Imager® Gel Doc™ XR System (Bio-Rad, USA).

### qRT-PCR Experiment

Gill tissues from 15 *C. gigas* and 15 *C. angulata* were collected for total RNA extraction using the TRIzol reagent (Solarbio). First-strand cDNA was obtained using HiScript III RT SuperMix for qPCR (Vazyme Biotech). The primers (supplementary table S2, Supplementary Material online) were designed using Primer 5 software and synthesized by Tsingke Biotechnology. Each group including three biological replicates, was mixed equally from cDNA of five oysters, and conducted three technical replicates. The *Ef-1α* gene was used as an internal control. qPCR was performed using the Taq Pro Universal SYBR qPCR Master Mix (Vazyme Biotech). Relative expression levels were calculated using the 2^−△△CT^ method (Livak and Schmittgen 2001). A detailed comparison between the two species has been previously described (Li et al. 2019; Wang, Li, Wang, Cong, et al. 2021).

### Luciferase Reporter Assay

The *Scd cis*-regulatory region of *C. gigas* (2,461 bp) and *C. angulata* (2,449 bp) was generated by PCR and constructed into the pGL3-basic vector (MiaoLing Plasmid Platform) using the ClonExpress II One Step Cloning Kit (Vazyme Biotech). The primers used to amplify fragments of the *Scd cis*-regulatory region are listed in supplementary table S3, Supplementary Material online. *Hin*dIII site was selected to construct the vector (New England Biolabs). Cell culture was performed as described and transfected with 480 ng (per well) of plasmids containing the *Scd cis*-regulatory region of *C. gigas* and *C. angulata* and 20 ng (per well) of the pRL-TK Renilla luciferase plasmid (MiaoLing Plasmid Platform) using Lipofectamine 3000 (Invitrogen). Luciferase activity was determined using the Dual-Luciferase Reporter Assay System (Promega, USA) and measured using Varioskan Flash multimode reader (Thermo Fisher Scientific). All experiments were conducted three technical replicates, and the firefly luciferase activity was normalized to the Renilla luciferase activity of each sample.

### Screening and Identification of the Causative Genetic Mutations

We compared the sequences within the 2.8-kb *Scd cis*-regulatory region between 15 *C. gigas* and 15 *C. angulata* using mixed-pool target amplicon sequencing. Primers for the amplification of the *Scd* promoter were designed using Primer 5 and synthesized by Tsingke Biotechnology (supplementary table S3, Supplementary Material online). All PCR products were amplified using 2 × Phanta Max Master Mix (Vazyme Biotech). The 20-μl reaction volume included 2 μl DNA as a template, 10 μl 2 × Phanta Max Master mix, 2 μl primers, and 6 μl H_2_O. The reaction conditions were as follows: 3 min of denaturation at 95 °C, followed by 35 cycles of amplification (95 °C for 15 s, 56 °C for 30 s, and 72 °C for 3 min), and a final extension of 5 min at 72 °C. The expected sizes of the DNA products were confirmed by agarose gel electrophoresis. The DNA products were purified using the FastPure Gel DNA Extraction Mini Kit (Vazyme Biotech). The concentration of DNA products was determined using NanoDrop 2000 and Qubit 2.0, and the same amounts of *C. gigas* and *C. angulata* PCR products were mixed into a separate pool (1 pool mixed with 15 oyster DNA samples), respectively. Two pooled DNA samples were generated: Ca (*C. angulata*) and Cg (*C. gigas*). The genotypes of the variants were identified by Tsingke Biotechnology. The mixed DNA samples were broken into fragments of ∼350 bp using a Covaris M220 ultrasonicator (Covaris, USA). DNA libraries were generated using the NEBNext Ultra DNA Library Prep Kit for Illumina (New England Biolabs). Briefly, the DNA samples were end-polished, A-tailed, and ligated with the full-length adapter for Illumina sequencing, then amplified by PCR, and purified using the AMPure XP system (Beckman Coulter, USA), and DNA concentration was measured using a Qubit® 3.0 Fluorometer (Invitrogen), libraries were analyzed for size distribution by Agilent 2100 Bioanalyzer and quantified by RT-PCR (>2 nM), and sequenced on an Illumina Novaseq 6000 platform and paired-end reads were generated. To obtain high-quality clean reads, the adapter sequences were filtered, and reads with >10% unknown nucleotides (N), and reads with >40% low-quality based (*Q* ≤ 15) were removed using Fastp (version 0.20.1) with default parameters (Chen et al. 2018). Then, clean reads were mapped against the reference sequences (GenBank accession no. ON220178) using the Burrow-Wheeler Aligner (Li and Durbin 2009) with default parameters. SNP calling was performed using HaplotypeCaller in GATK (version 3.4; Van der Auwera et al. 2013). The *χ*^2^ test was performed based on site sequencing depth to screen for significantly different sites between the two groups. Based on the results of genotyping using mixed-pool target amplicon sequencing, the variation sites were further confirmed by Sanger sequencing. The DNA of 50 wild *C. gigas* and 50 wild *C. angulata* was extracted using the TIANamp Marine Animals DNA Kit (TIANGEN BIOTECH, China). The primers were designed using Primer 5 and synthesized by Tsingke Biotechnology (supplementary table S3, Supplementary Material online). The reaction was the same as that described above. PCR products were sequenced by Tsingke Biotechnology after agarose gel electrophoresis and gel extraction. The sequences were aligned to the reference sequence (GenBank accession No. ON220178) and genotyped using VectorNTI (version 8.0) (Invitrogen). Linkage disequilibrium analysis (D” value) was calculated using SHEsis (Shi and He 2005).

### Functional Analysis of Mutations

Every variation site was mutated using the pGL3-*CgScd* plasmid using the Mut Express® II Fast Mutagenesis Kit V2 (Vazyme Biotech). The primers were synthesized by Tsingke Biotechnology (supplementary table S3, Supplementary Material online). The 50-μl reaction volume included 2 μl pGL3-*CgScd* as a template, 1 μl Phanta Max Super-Fidelity DNA Polymerase, 4 μl primers, 1 μl dNTP Mix, 25 μl 2 × Max Buffer, and 17 μl H_2_O. The reaction procedure was as follows: 30 s of denaturation at 95 °C, followed by 35 cycles of amplification (95 °C for 15 s, 60 °C for 15 s, and 72 °C for 8 min) and a final extension of 5 min at 72 °C. After the expected size of PCR products was confirmed by agarose gel electrophoresis, 1 μl *Dpn*I was added to purified PCR products, then incubated for 2 h at 37 °C. Finally, the plasmids were restructured according to the following reaction: 4 μl 5 × CE II buffer, 2 μl Exnase II, 6 μl incubation products, and 8 μl H_2_O. The mixture was incubated for 30 min at 37 °C. Then, the single-site mutation plasmids were transformed into DH5α competent cells (Tsingke Biotechnology) to confirm the desired mutation by DNA sequencing. The luciferase reporter assay procedure was the same as that described above.

### DNA Pull-Down Assay

DNA pull-down was performed according to a previous study with a slight modification (Gabrielsen et al. 1989). Nuclear extracts were prepared from *C. gigas* gill tissues using NE-PER Nuclear and Cytoplasmic Extraction Reagents (Thermo Fisher Scientific). The extraction of nuclear proteins was confirmed by western blotting with anti-histone H3 antibody (Abcam). The biotin-labeled probe was designed by extending 20 bp at both ends of the selected SNPs and synthesized by Tsingke Biotechnology (supplementary table S4, Supplementary Material online). Double-stranded probes were generated by annealing the single-stranded complementary oligonucleotides. The probes were then immobilized onto streptavidin-coated magnetic beads (Thermo Fisher Scientific) in a DNA-binding buffer (10 mM Tris-HCl, 1 mM EDTA, and 2 M NaCl; pH 7.5). The control group contained empty magnetic beads. The beads were resuspended in protein-binding buffer (20 mM Tris-HCl, 4.5 mM EDTA, 60 mM NaCl, 10 mM HEPES, 5 mM CaCl_2_, 50 mM KCl, 9% sucrose (w/v), and 12% glycerol; pH 7.5). Nuclear protein extracts were then added and the mixture was incubated at room temperature for 15 min. The magnetic particles were washed thrice with protein-binding buffer, and the proteins were eluted with elution buffer (25 mM Tris-HCl and 200 mM NaCl; pH 7.5). Eluted proteins were analyzed by SDS-PAGE and sliver staining. All individual lanes were identified by MS as follows.

The bands were excise and cut into 0.5–0.7 mm pieces. After destaining with the test stain decolorizing solution, the gel pieces were washed thrice with 500 μl of acetonitrile solution until the rubber particles were completely white. Next, 500 μl of 10 mM dithiothreitol was added and incubated into water bath at 56 °C for 30 min, followed by centrifugation, and the supernatant was discarded. The gel pieces were incubated with 500 μl of decolorizing solution at room temperature for 5–10 min, washed once, and centrifuged to discard the supernatant. Then, 500 µl of 55 mM iodoacetamide were added and the mixtures were placed in the dark at room temperature for 30 min, centrifuged to discard supernatant, then incubated with 500 μl of decolorizing solution at room temperature for 5–10 min. The gel pieces were washed once and centrifuged to discard the supernatant. Acetonitrile (500 μl) was added until the gel pieces were completely white, which were then vacuum dried for 5 min. Trypsin (0.01 μg) was added and the mixture was placed in an ice bath for 30 min. To enzymatically hydrolyze the gel pieces, 25 mM NH_4_HCO_3_ enzymatic hydrolysis buffer (pH 8.0) was added and the mixture was incubated overnight at 37 °C. The next day, 300 μl of the extract liquor was added, the mixture was sonicated for 10 min and centrifuged, and the supernatant was collected. After repeating twice, the obtained extracts were combined and vacuum dried. The samples were dissolved with 10–20 μl of 0.2% trifluoroacetic acid and centrifuged at 10,000 g for 20 min. The Ziptip was washed 15 times with a wetting solution and was equilibrated by aspirating and discarding the equilibration and sample solutions 10 times. Then, the column was washed by aspirating and discarding the rinse liquid eight times. Fifty microliters of eluent were added to a clean tube and pipetted repeatedly to elute the peptide, and the samples were drained. The samples were diluted to 1 μg/μl in a buffer. The sample volume was set to 5 μl and the scan mode was performed for 60 min. The peptides were scanned with a mass-to-charge ratio of 350–1200 in the samples, and the MS data were collected using a Triple TOF 5600 + LC/MS system (AB Sciex, USA). The samples were dissolved in 2% acetonitrile/0.1% formic acid and analyzed using a Triple TOF 5600 plus mass spectrometer coupled with an Eksigent nanoLC system (AB Sciex). Then, the solution was added to a C18 capture column (3 μm, 350 μm × 0.5 mm; AB Sciex), and a C18 analytical column (3 μm, 75 μm × 150 mm; Welch Materials, USA) was applied with a 60-min time gradient and a flow rate of 300 nl/min for gradient elution. The buffer A (2% acetonitrile/0.1% formic acid/98% H_2_O) and buffer B (98% acetonitrile/0.1% formic acid/2% H_2_O) consisted the two mobile phases. The MS spectrum was scanned with an ion accumulation time of 250 ms, and the MS spectrum of 30 precursor ions was acquired with an ion accumulation time of 50 ms for information-dependent acquisition. The MS1 spectrum was collected in the range of 350–1200 *m*/*z*, and the MS2 spectrum was collected in the range of 100–1500 *m*/*z*. The precursor ion dynamic exclusion time was set to 15 s. The original MS/MS files from the mass spectrometer were submitted to ProteinPilot (https://sciex.com.cn/products/software/proteinpilot-software, version 4.5; AB Sciex) for data analysis. The Paragon algorithm in ProteinPilot was used to identify proteins by searching the *C. gigas* protein database (Qi et al. 2021). The parameters were set as follows: the instrument was TripleTOF 5600, cysteine was modified with iodoacetamide, and biological modification was selected as the ID focus. For the identified protein results, certain filtering criteria were applied, peptides with an unused score >1.3 (credibility >95%) were considered credible peptides, and proteins containing at least one unique peptide were retained. Potential regulatory proteins interacting with the mutation sites were screened by comparing the two probe groups and the control group.

### Electrophoretic Mobility Shift Assay

The recombinant truncated *Cg*YB containing an N-terminal 6×His-tag was expressed using pET-28a-SUMO (MiaoLing Plasmid Platform) and *Escherichia coli* BL21 (DE3; Tsingke Biotechnology). The ORF of *Cg*YB was truncated (63–131 aa) based on domain prediction by SMART (http://smart.embl-heidelberg.de/; Letunic et al. 2021) and disordered residues predicted by IUPred3 (https://iupred.elte.hu/; Erdős et al. 2021; supplementary table S3 and fig. S3, Supplementary Material online). The PCR product was ligated into the *Sac*I and *Xho*I restriction sites of the pET-28a-SUMO vector (New England Biolabs). The recombinant plasmid was transformed into *E. coli* (DE3). The transformant was grown in LB medium at 37 °C to an OD600 of 0.6. Isopropyl β-D-1-thiogalactopyranoside (1 mM; Solarbio) was added to the culture for 16 h growth at 16 °C. Truncated *Cg*YB was purified using Ni-NTA agarose (ComWin Biotech, China) according to the manufacturer's instructions. Purified truncated *Cg*YB was desalted, concentrated, and replaced with phosphate-buffered solution (PBS) as buffer solution using Pierce™ Protein Concentrators (Thermo Fisher Scientific). The recombinant proteins were analyzed using 12% SDS-PAGE (GenScript Biotech) and stained with Coomassie Brilliant Blue R-250. The protein concentration was determined using an Enhanced BCA Protein Assay Kit (Shanghai Epizyme Biomedical Technology, China). The selected SNP was functionally characterized using EMSA to identify its potential to affect DNA–protein interactions. The DNA probes labeled with 5′ biotin were synthesized by Tsingke Biotechnology, and biotinylated and unlabeled probe sequences were as follows: F, 5′-AAGGAGTGAC**C**ATCCCTTGCA-3′; mutated probe: F, 5′-AAGGAGTGAC**T**ATCCCTTGCA-3′. Double-stranded probes were generated by annealing the single-stranded complementary oligonucleotides. EMSA experiments were performed using the LightShift® Chemiluminescent EMSA Kit (Thermo Fisher Scientific), according to the manufacturer's instructions. For each binding reaction containing 2 μl binding buffer, 1 μl poly(dI:dC), 10 μl ddH2O, and 3 μl labeled probes (1 pmol/μl), 4 μl truncated *Cg*YB (1 mg/ml) was added. Unlabeled probes (3μl; 10 pmol/μl) was added in the cold competition group. After the mixtures were incubated for 30 min at 25 °C, the DNA–protein complexes were separated by electrophoresis on a 6% nondenaturing polyacrylamide gel at 120 V for 40 min in a cold 0.5X Tris-Borate-EDTA (TBE) running buffer. The separated complexes were transferred to a nylon membrane (Millipore) at 100 V for 1 h in cold 0.5X TBE. The DNA–protein complexes were crosslinked using a transilluminator with UV bulbs. Biotinylated probes were detected using the Molecular Imager Gel Doc XR System (Bio-Rad). The position frequency matrix of the Y-box factor-binding motif was downloaded from the JASPAR database (https://jaspar.genereg.net/) (Fornes et al. 2020) and redrawn using TBtools (Chen et al. 2020).

### Short-Term Cold Stress Experiment

Oysters from 30 *C. gigas* and 30 *C. angulata* were cleaned and reared in a 500-l tank for 1 week for acclimatization. On average 4 g/m^3^ commercial Spirulina powder was fed once per day (Wang, Li, Wang, Cong, et al. 2021). After the acclimation period, oysters were placed into 4 °C sand-filtered seawater for 10 days (15 *C. gigas* and 15 *C. angulata*). The temperature was controlled using a water bath with a temperature-controlled system. The control group (15 *C. gigas* and 15 *C. angulata*) was cultured in seawater at room temperature (18 ± 2 °C). The gills were sampled, and placed in liquid nitrogen, and stored in a −80 °C refrigerator for qRT-PCR and fatty acid measurement.

### Measurement of Fatty Acids Content

Gas chromatography was used to measure the fatty acid content of the gill tissues from three replicates that were mixed from five oysters during short-term cold adaptation. The protocol was same with our previous study (Wang, Li, Wang, Cong, et al. 2021): C19:0 fatty acid methyl ester (Sigma-Aldrich) and 0.01% butylhydroxytoluene methanol solution was added to sample for internal standard and antioxidant, respectively. The total fat was extracted using dichloromethane:methanol, and the sample was dried using high-purity nitrogen. Then, 0.5 M KOH methanol solution (1 ml) was added to the mixture, and saponified in a water bath at 80 °C for 2 h under the protection of nitrogen. After cooling, 14% BF3 methanol solution (1 ml) was added to the sample, and samples were incubated in a water bath at 80 °C for 1 h for methyl esterification reaction. Fatty acid methyl esters were extracted using n-hexane. The sample volume was adjusted to 0.5 ml, and analyzed using an Agilent 7890A gas chromatograph (Agilent Technologies, USA). The chromatographic conditions were as follows: capillary column: DB-FFAP (30 m * 0.32 mm * 0.25 μm); inlet temperature: 220 °C; detector temperature: 280 °C; column temperature: program heating 150 °C (1 min), 3 °C/min and 220 °C (33 min).

### Identification of Regulatory Relationships

Genome annotation of *C. gigas* showed that only *Ppar* and *Srebp* are present in oysters. To determine the transcriptional activity of SREBP on oyster *Scd*, pcDNA3.1-*Cg*SREBP and pcDNA3.1-myc-HisA plasmid were transfected into the HEK293T cells. Additionally, pGL3-basic carrying the *Scd* promoter fragment and pRL-TK were co-transfected to determine the regulatory relationship. The control group was transfected with pRL-TK, pcDNA3.1-myc-HisA, and pGL3-*CgScd* or pGL3-*CaScd*, whereas the sample group was transfected with pRL-TK, pcDNA3.1-*Srebp* and pGL3-*CgScd* or pGL3-*CaScd*. The luciferase reporter assay procedure was the same as that described above. Cells were collected for protein extraction using the M-PER™ Mammalian Protein Extraction Reagent (Thermo Fisher Scientific). Western blotting was performed to demonstrate the protein expression, as described above. The protein purification procedure was the same as described above. The ORF of *CgSrebp* was truncated (1–460 aa) due to the prediction of protein domain by SMART (http://smart.embl-heidelberg.de/; Letunic et al. 2021) and the prediction of transmembrane region by TMHMM 2.0 (https://services.healthtech.dtu.dk/service.php?TMHMM-2.0; the details of primers are shown in supplementary table S3 and fig. S4, Supplementary Material online). The EMSA was performed as described. The potential SREBP binding motif was predicted using AnimalTFDB 3.0 (Hu et al. 2018). The DNA probes labeled with 5′ biotin were synthesized by Tsingke Biotechnology, and the biotinylated and unlabeled probe sequences were: F, 5′-AGAATCGCGTGAAAA-3′. The position frequency matrix of the SREBP binding motif was downloaded from JASPAR (https://jaspar.genereg.net/) (Fornes et al. 2020) and redrawn using TBtools (Chen et al. 2020).

### Statistical Analysis

All statistical analyses were performed using GraphPad Prism version 8.0.2 for Windows. Statistical analyses were conducted after confirming the normality of the distributions using the Shapiro–Wilk test and homogeneity of variance using Bartlett's test. Comparisons between two groups were performed by two-tailed unpaired Student's *t*-test, and one-way analysis of variance (ANOVA) followed by Tukey’s multiple comparisons test were used for comparisons among three groups or more. Among these, comparisons of relative gene expression during short-term cold stress were performed using two-way ANOVA. Significant differences between groups were marked with “*” for *P* < 0.05, “**” for *P* < 0.01, and “***” for *P* < 0.001.

## Supplementary Material

msad015_Supplementary_Data

## Data Availability

The cDNA sequence of *CgScd* was deposited in the GenBank with accession number ON220179. And the promoter sequence of oyster *Scd* in *C. gigas* and *C. angulata* was deposited in the GenBank with accession numbers ON220178 and ON220180, respectively.
