# Supplementary material for: Cis- and Trans-variations of Stearoyl-CoA Desaturase Provide New Insights into the Mechanisms of Diverged Pattern of Phenotypic Plasticity for Temperature Adaptation in Two Congeneric Oyster Species
Source: Mol Biol Evol. 2023 Jan 20;40(2):msad015. doi: 10.1093/molbev/msad015 (PMC9949715; doi:10.1093/molbev/msad015)
Supplement: msad015_Supplementary_Data [file msad015_supplementary_data.zip › Supplemental_file.docx]

**Supplemental File**

Supplementary figures


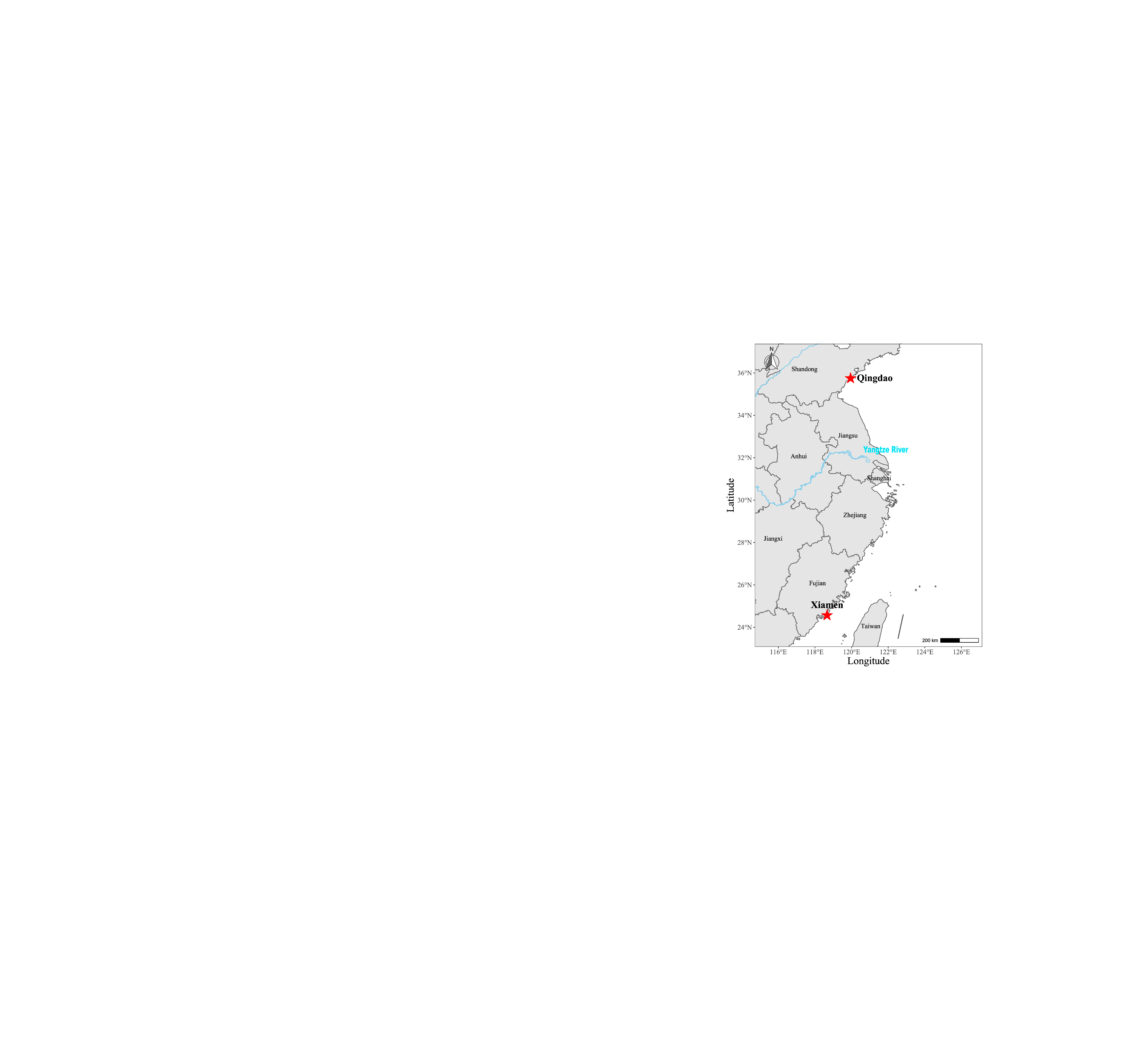


Supplementary Fig. 1 Collection sites of wild *Crassostrea gigas* and *Crassostrea angulata* from northern (Qingdao) and southern (Xiamen) of China.

**
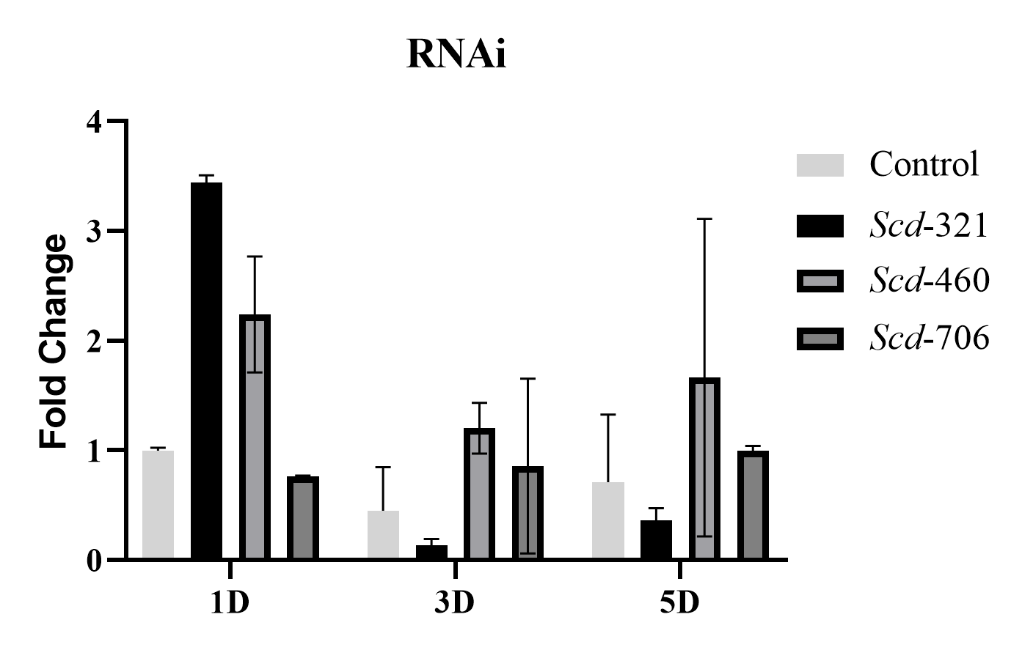
**

Supplementary Fig. 2 The results of the pilot experiment of RNAi. A total of three small RNA interference strands were tested by measuring the expression level of *Scd*. And the results showed that the *Scd*-321 and 3 d (72 h) were the most effective siRNA and time. The qRT-PCR were taken using the cDNA of the *C.gigas* gill which were injected with the siRNA after anesthetized with the MgCl2. The horizontal axis represents time of the 1, 3, 5 days from the experiment start and the legend shows the name of each small RNA, including *Scd*-321, *Scd*-460 and *Scd*-706.


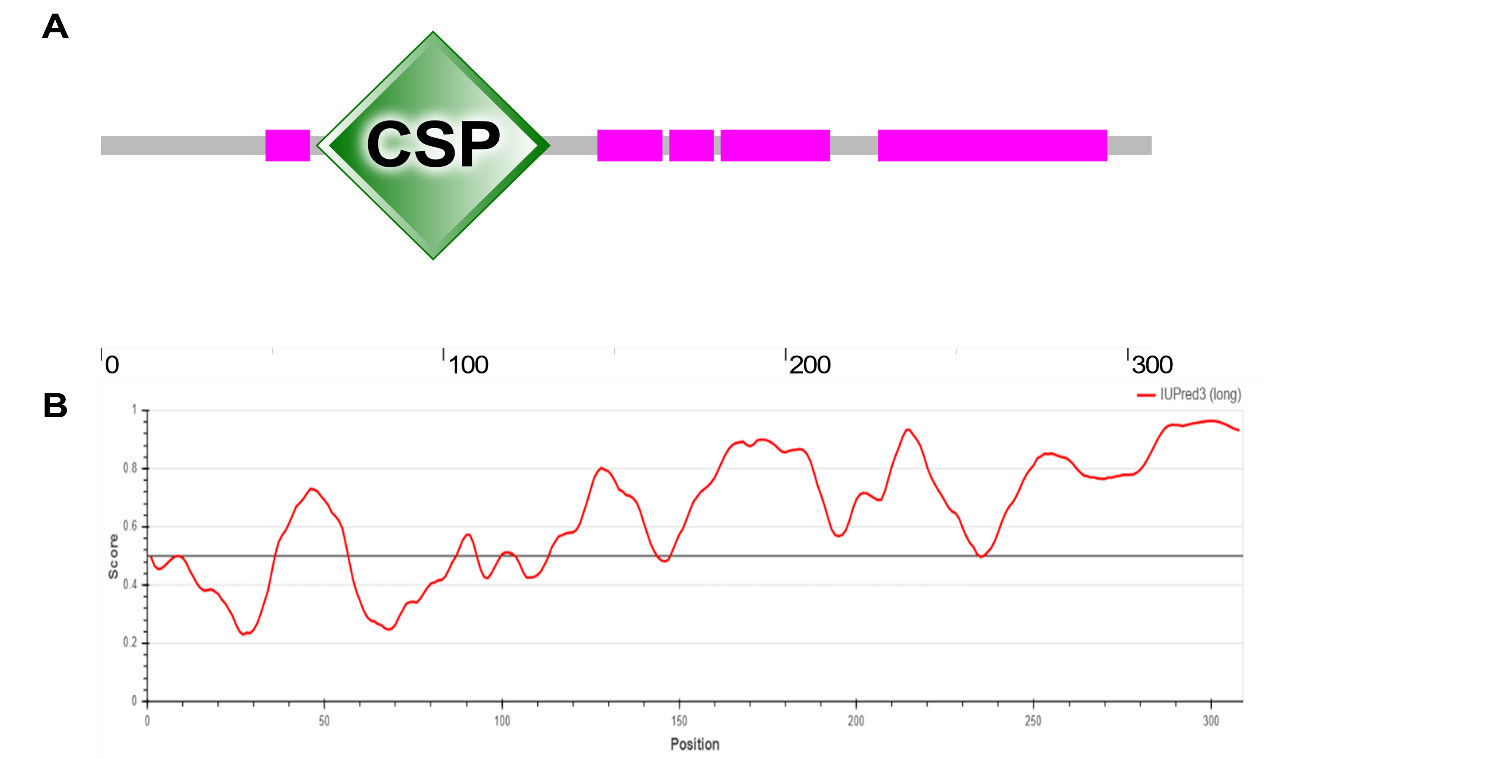


Supplementary Fig. 3 The characterization of *Cg*YB protein sequence. (A) The statistics of *Cg*YB domain prediction by SMART (<http://smart.embl-heidelberg.de/>) (Letunic, et al. 2021). The results showed that 63 to 131 aa of *Cg*YB is the ‘Cold-shock’ DNA-binding domain. (B) The statistics of disordered residues of *Cg*YB by IUPred3 (<https://iupred.elte.hu/>) (Erdős, et al. 2021). The results showed that most of the sequence of *Cg*YB is disordered residues (Score > 0.5) except. Therefore, we truncated the ORF of *Cg*YB (61-131 aa) to remove the disordered residues and maintain its DNA binding domain.


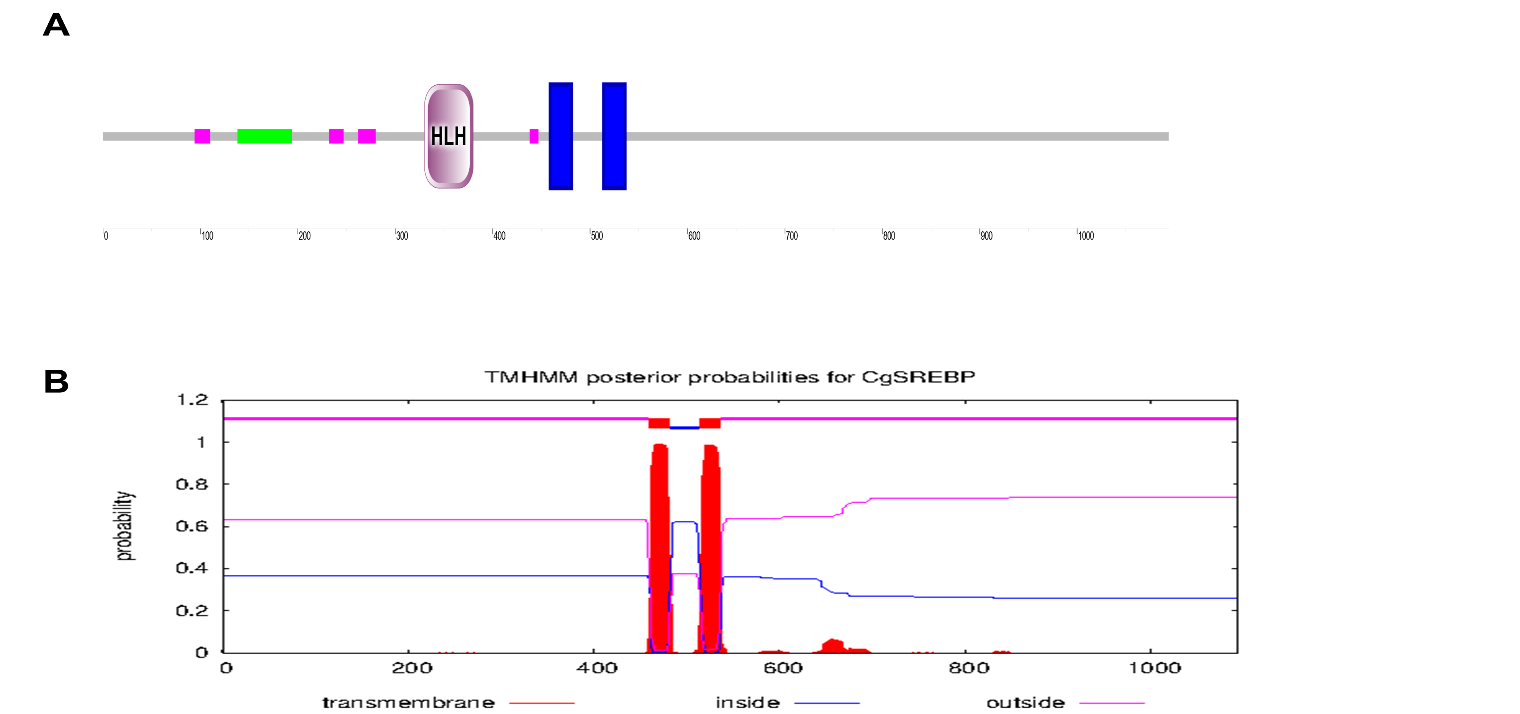


Supplementary Fig. 4 The characterization of *Cg*SREBP protein sequence. (A) The statistics of *Cg*SREBP domain prediction by SMART (<http://smart.embl-heidelberg.de/>) (Letunic, et al. 2021). The results showed that 330 to 380 aa of *Cg*SREBP is the Helix-Loop-Helix-zipper (bHLHzip) domain, which is used for recognizing sterol regulatory element (SRE), then activate the downstream genes transcription. (B) The statistics of transmembrane region of *Cg*SREBP by TMHMM 2.0, (<https://services.healthtech.dtu.dk/service.php?TMHMM-2.0>). The results showed that the *Cg*YB has two transmembrane region (459-481 aa, 514-536 aa). Therefore, we truncated the ORF of *Cg*SREBP was (1-460 aa) to removing the transmembrane region and maintain its DNA binding domain.


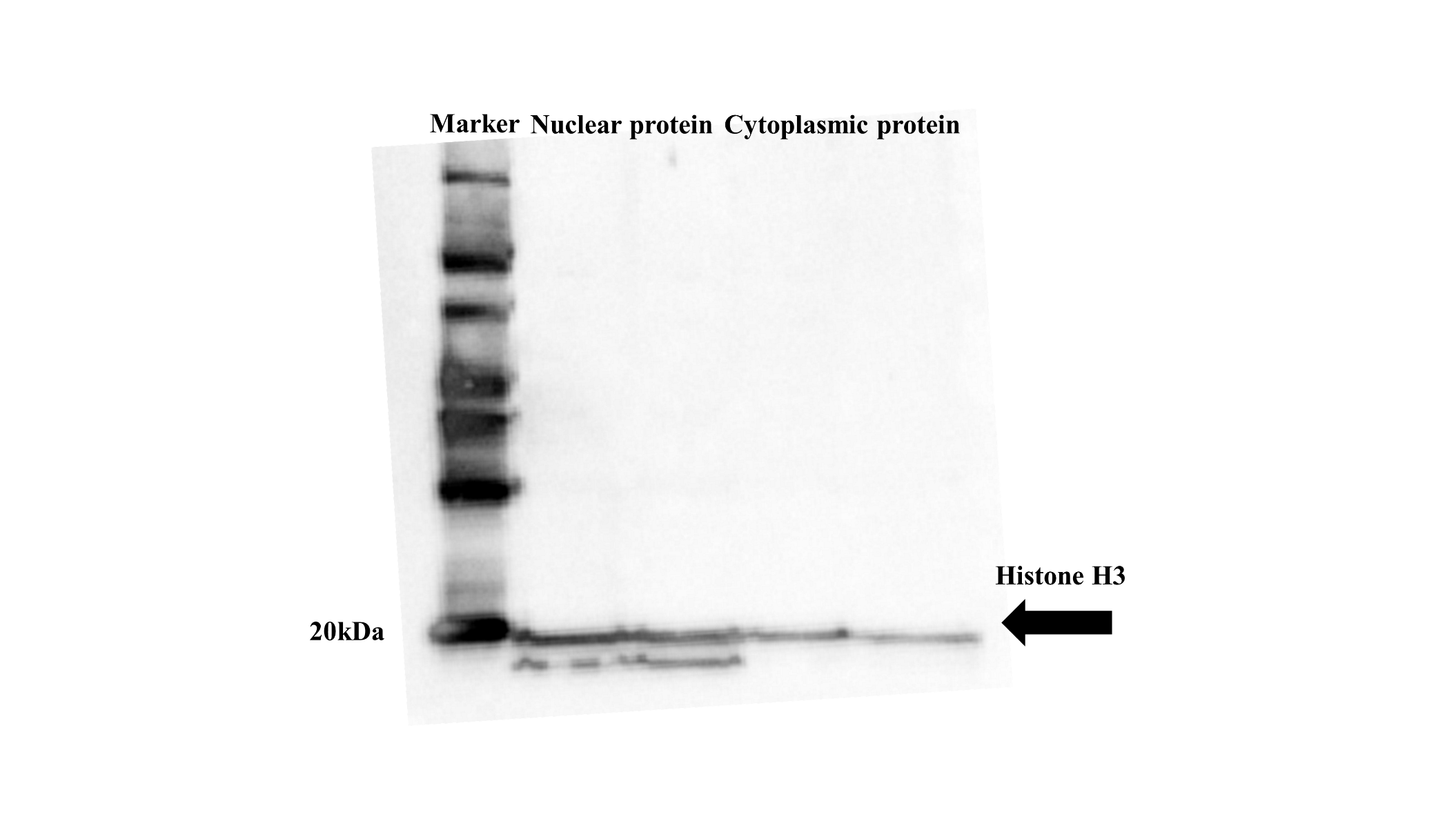


Supplementary Fig. 5 The western blotting results of oyster nuclear protein extraction quality control. The first lane in protein loading marker. The second and third lanes were the extracted oyster nuclear proteins. And the fourth and fifth lanes were the extracted cytoplasmic proteins. The black arrow represents the position of nuclear protein marker (histone H3). The results showed that clearer bands in the nuclear protein groups than cytoplasmic protein groups, which indicated high quality of extraction of nuclear proteins.


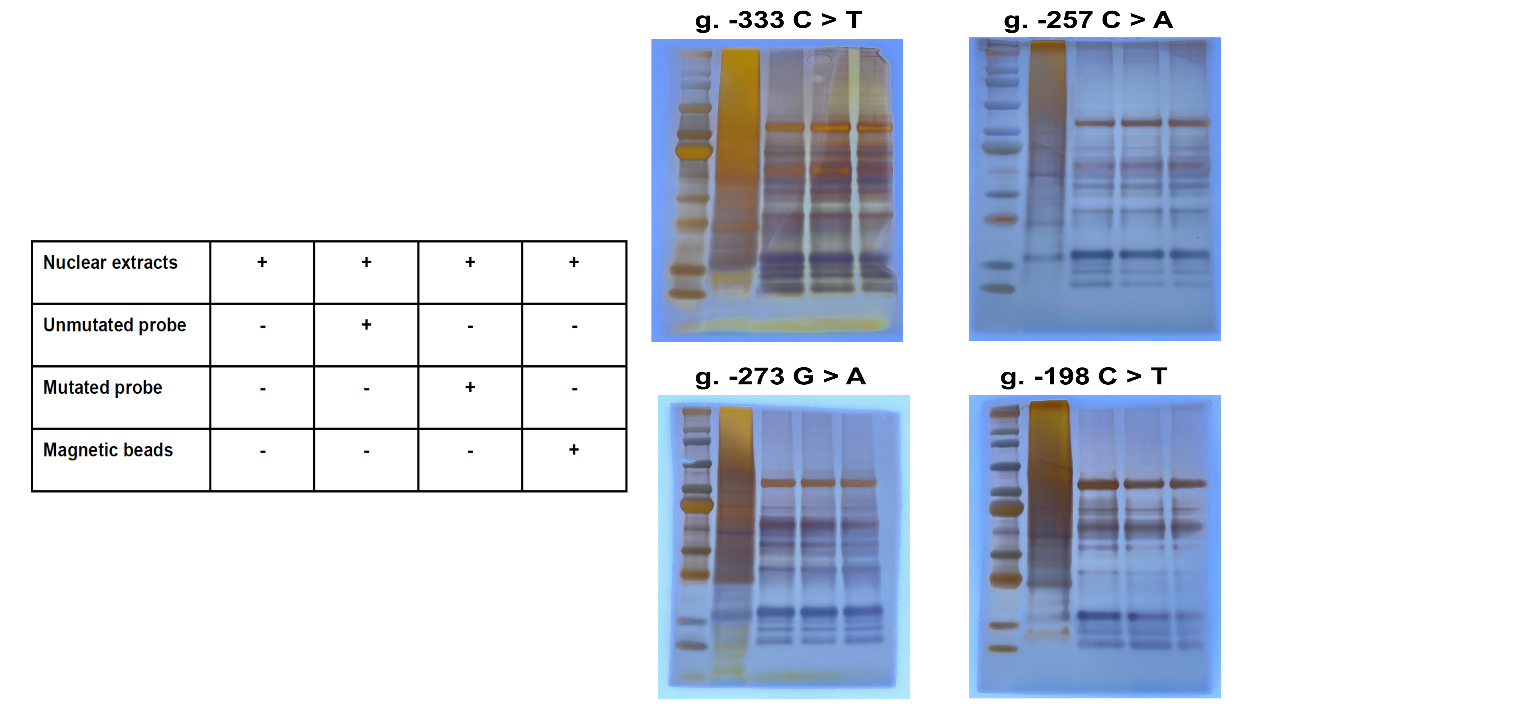


Supplementary Fig. 6 The sliver staining plot of DNA pull-down experiments. The first lane is the protein loading marker. The second lane is the reaction of nuclear extracts and unmutated probe. The third lane is the reaction of nuclear extracts and mutated probe. The fourth lane is the reaction of nuclear extracts and empty magnetic beads without biotin-labeled probe. Overall, binding proteins were consistently isolated across lanes, and there were no significantly different proteins.


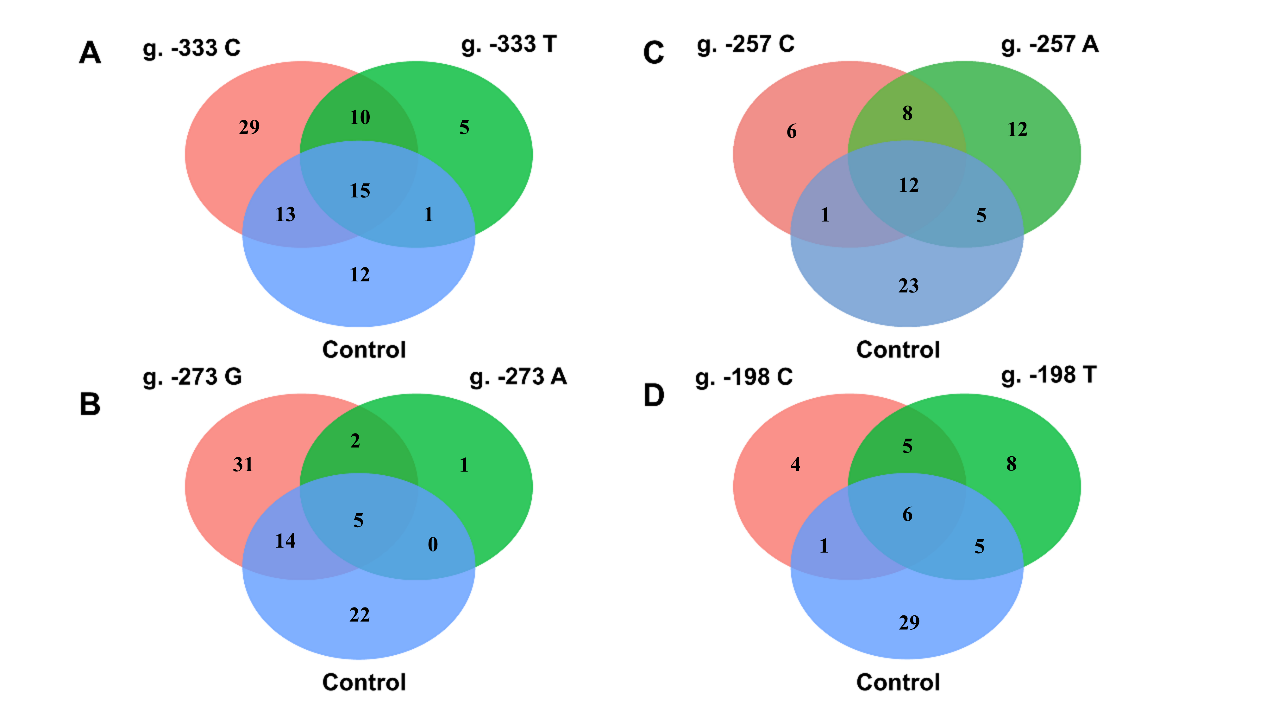


Supplementary Fig. 7 The Venn plot of MS analysis of DNA pull-down protein.


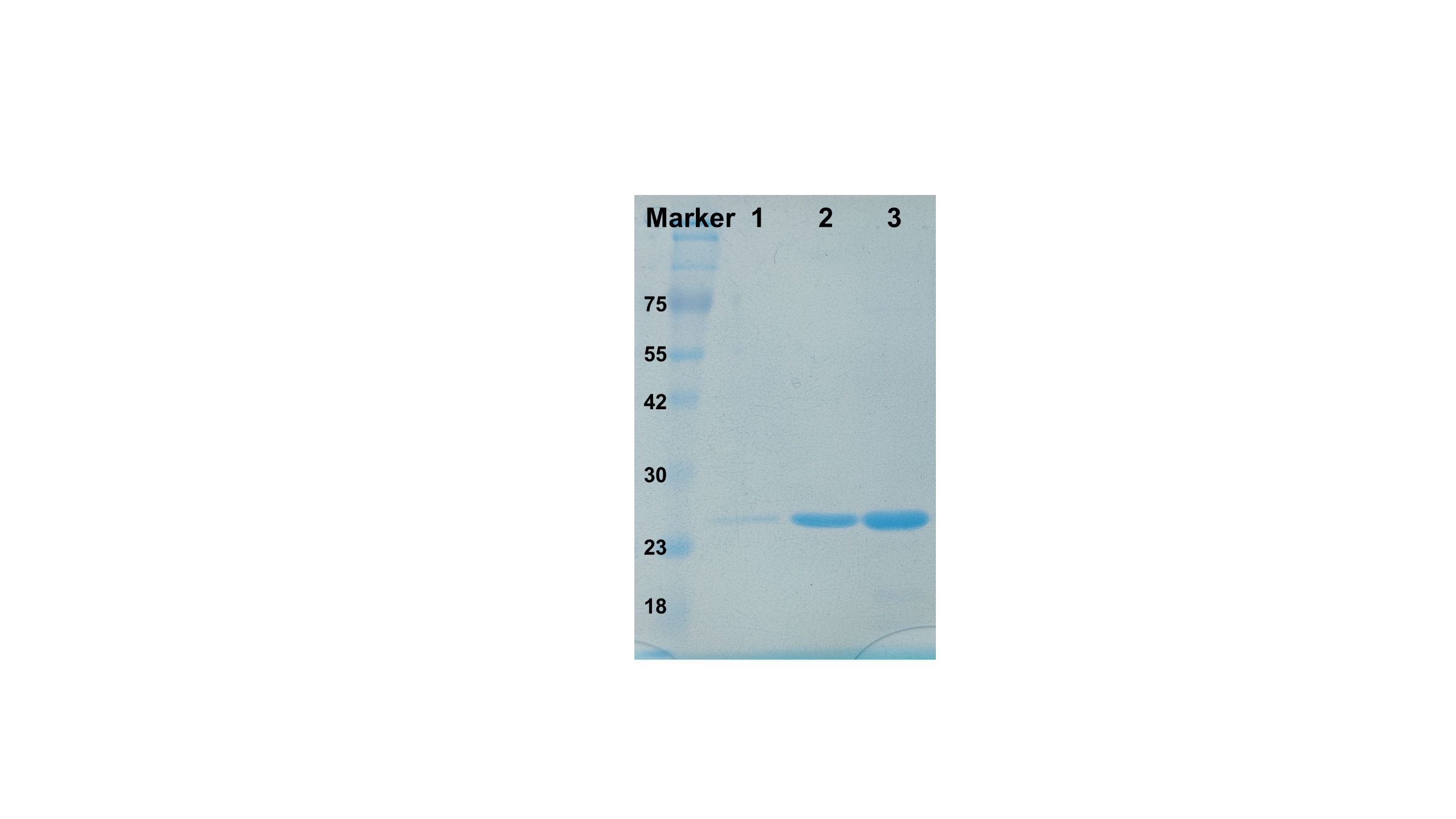


Supplementary Fig. 8 The purification of the truncated His_6_*-Cg*YB protein. The first lane is the protein loading marker. The second and third lanes are the purified truncated *Cg*YB protein eluted by 100% imidazole. And the third lane is the purified truncated *Cg*YB protein eluted by 30% imidazole. Black arrow represents the position of truncated His_6_-*Cg*YB, which met the predicted molecular weight (25kD).


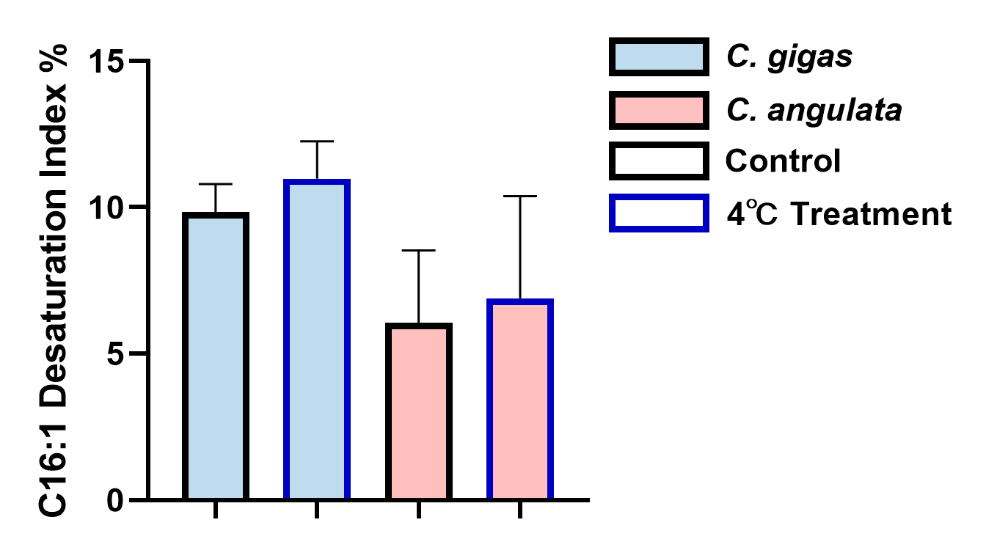


Supplementary Fig. 9 The palmitoleic acid (C16:1) desaturation index in *C. gigas* and *C. angulata* during short-term cold stress. The red and blue color represented the *C. gigas* and *C. angulata*. And the dark blue and black border represented the cold stress groups and control groups.


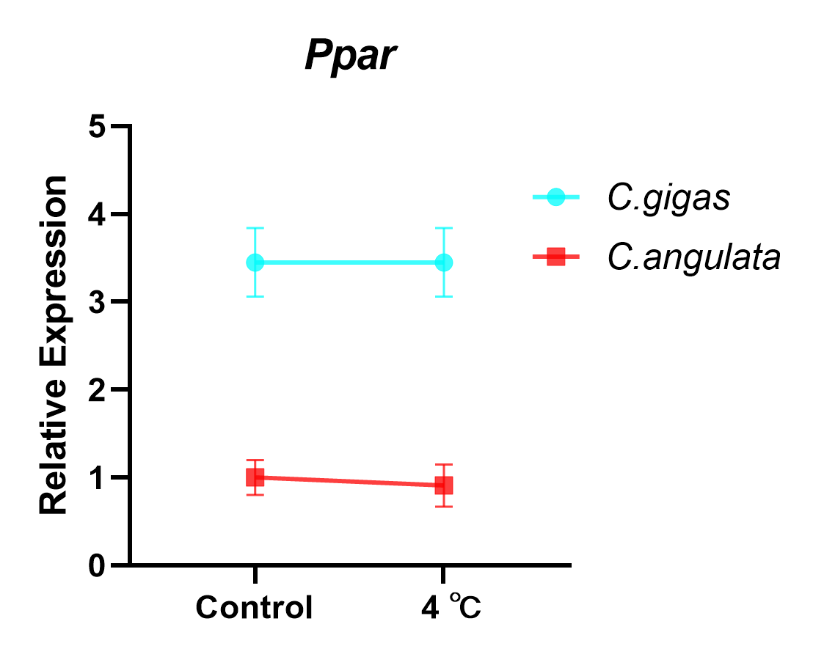


Supplementary Fig. 10 The relative gene expression of oyster *Ppar* during short-term cold stress. The red line represented *C. angulata*, and the blue line represents the *C. gigas*.


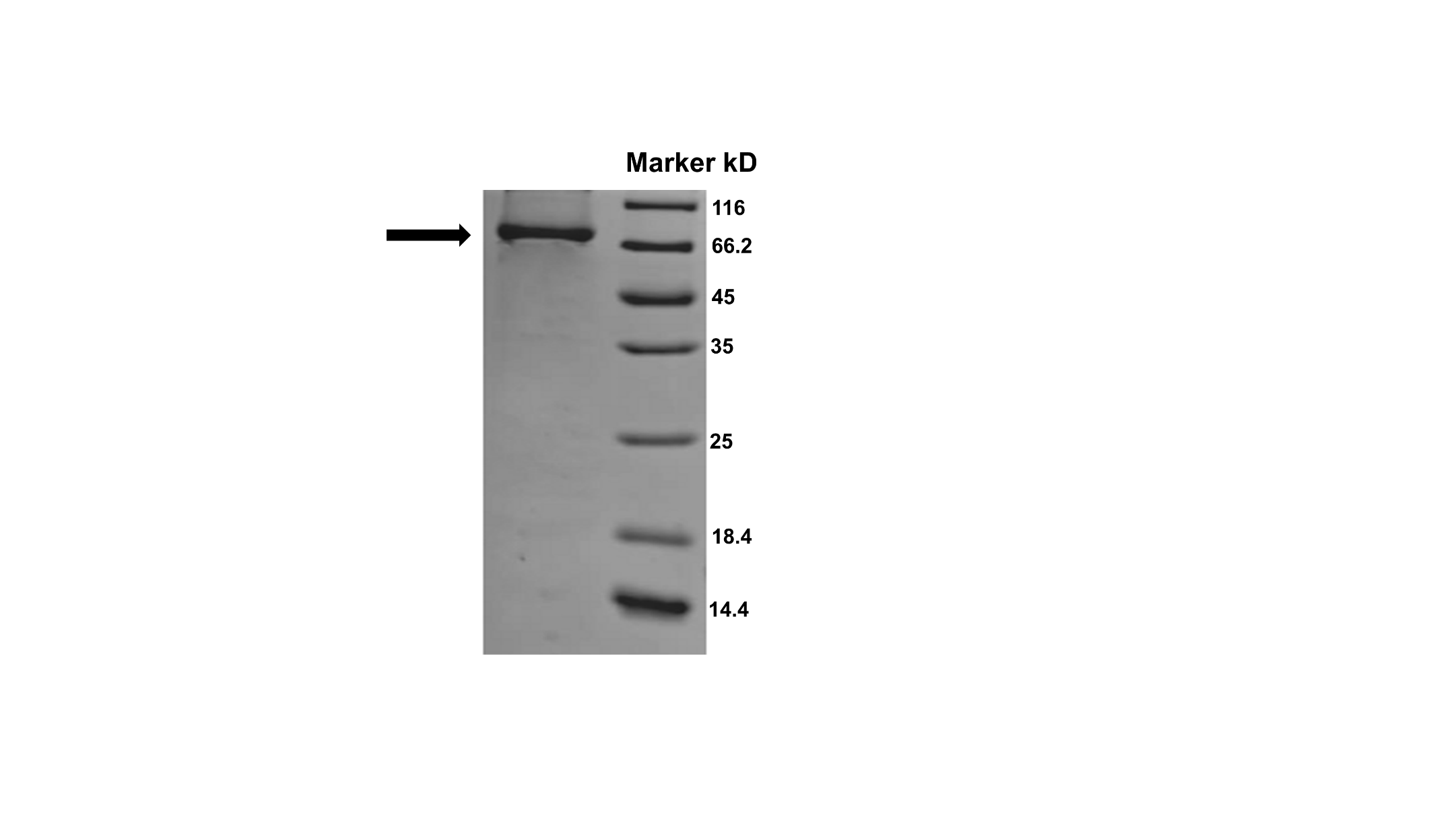


Supplementary Fig. 11 The purification of the truncated His_6_*-Cg*SREBP protein. The second lane is the protein loading marker and the black arrow indicated the position of truncated His_6_-*Cg*SREBP protein. The predicted molecular weight of the truncated *Cg*SREBP was 67kd, which met the expectation.

Supplementary Tables

| Names | Sense(5’-3’) | Antisense(5’-3’) |
| --- | --- | --- |
| *Scd*-321 | CUGCUCUCCAGAAUGAUAUTT | AUAUCAUUCUGGAGAGCAGTT |
| *Scd*-460 | CCCGGAUGUAAUGGAGAAATT | UUUCUCCAUUACAUCCGGGTT |
| *Scd*-706 | CAGGAAACCUUACGACAAATT | UUUGUCGUAAGGUUUCCUGTT |

Supplementary Table 1 The sequence of siRNAs in the RNA interference experiments.

| **Abbreviation** | **Primer sequence(5′-3′)** |
| --- | --- |
| ***Scd*** | **F: GTATTCCGAAACAGACGC** |
|  | **R: CACAGTAGGGACCACAAA** |
| ***Ef-1α* (*C.gigas*)** | **F: AGTCACCAAGGCTGCACAGAAAG** |
|  | **R: TCCGACGTATTTCTTTGCGATGT** |
| ***Ef-1α* (*C.angulata*)** | **F: TTCCCAGCAAGCCTATGT** |
|  | **R: GCTCAGCCTTCTCAACCTC** |
| ***Ppar*** | **F: CTGTGCGATGAACTACGA** |
|  | **R: AGAGTGTTTGCGATTTGA** |
| ***Srebp*** | **F: AGGACCTTCACAGTCAGCA** |
|  | **R: CTCATCTCCAGCCAACCA** |

Supplementary Table 2 Primer sequences for qRT-PCR.

| **Purpose** | **Primer sequence(5′-3′)** | **Purpose** |
| --- | --- | --- |
| **pYES3-*Scd*-F** | **CCGCTCGAGTCTAGAGGGCCCATGGCGCCAAGAAATGTTGT** | **Overexpression experiment** |
| **pYES3-*Scd*-R** | **AGGCTTACCTTCGAAGGGCCCCGAGCTTCCGTCCCCCGT** |  |
| **pcDNA-*Scd*-F** | **GAGTCTAGAGGGCCCTTCGAAATGGCGCCAAGAAATGTTGT** | **Fluorescence Recovery After Photobleaching** |
| **pcDNA-*Scd*-R** | **TGAGATGAGTTTTTGTTCGAACGAGCTTCCGTCCCCCGT** |  |
| **pGL3-*CgScd*-F** | **ATCTGCGATCTAAGTAAGCTTTAGTTTCCAGAAGTGGATGGAGC** | **Luciferase reporter assay** |
| **pGL3-*CgScd*-R** | **CAGTACCGGAATGCCAAGCTTCGAGGTGAAGGGAAGACATGAG** |  |
| **pGL3-*CaScd*-F** | **ATCTGCGATCTAAGTAAGCTTTAGTTTCCAGAAGTGGATGGAGC** | **Luciferase reporter assay** |
| **pGL3-*CaScd*-R** | **CAGTACCGGAATGCCAAGCTTCGAGGTGAAGGGAAGACATAAGG** |  |
| **Cis-*Scd*-**  **F1** | **GAGGTGAAGGGAAGACATAAGGACGA** | **Genotyping by mixed pool target amplicon sequencing** |
| **Cis-*Scd*- R1** | **CGTATTGAAAGGGCGGCAGAT** |  |
| **Cis-*Scd*-**  **F2** | **GGCCAAGTGGATTACACTCG** | **Sanger sequencing** |
| **Cis-*Scd*- R2** | **TGCTAACAGTTTCGCTCCAC** |  |
| **-1207 C>A-F** | **GTAGACATTTGACTGCCTTTCGAACATGAACC** | **pGL3-*CgScd*:**  **-1207C>A Single Site Mutation** |
| **-1207 C>A-R** | **GGCAGTCAAATGTCTACTTATAGAGAAAGGTCGCTGAATT** |  |
| **g.-1196 T>C-F** | **TTGACTGCGTTTCGAACATGAACCATTCAGCG** | **pGL3-*CgScd*:**  **-1196T>C Single Site Mutation** |
| **g.-1196 T>C-R** | **GTTCGAAACGCAGTCAAATATCTACTTATAGAGAAAGGTC** |  |
| **g.-1185 C>G-F** | **CGGGGTAAAAACGTTCATACGTAAACATATATTGTGTCTTACGAC** | **pGL3-*CgScd*:**  **-1185C>G Single Site Mutation** |
| **g.-1185 C>G-R** | **TGAACGTTTTTACCCCGTGTTATAGAATCGAA** |  |
| **g.-1107 A>T-F** | **GTATAATCATTTTTGCTATAATATATATATTACAATACTACATATGAATACACATAGC** | **pGL3-*CgScd*:**  **-1107A>T Single Site Mutation** |
| **g.-1107 A>T-R** | **AGCAAAAATGATTATACATGTACATGTAAATACA** |  |
| **g.-908 T>C-F** | **ACATGCAGGTCGATTACAAAACTAACATCTAACTGTGG** | **pGL3-*CgScd*:**  **-908T>C Single Site Mutation** |
| **g.-908 T>C-R** | **GTAATCGACCTGCATGTACTACAAGTACGTAAATGC** |  |
| **g.-720 A>G-F** | **GTCTTTAAGTTAGCCCCATGTTAATATATTAT** | **pGL3-*CgScd*:**  **-720A>G Single Site Mutation** |
| **g.-720 A>G-R** | **GGGGCTAACTTAAAGACTATATAATTATATGTACAGATTCATAGATATAAACCG** |  |
| **g.-649 A>G-F** | **GGATAGCCCAAATCACATGGTGTAAAGAAAACCCA** | **pGL3-*CgScd*:**  **-649A>G Single Site Mutation** |
| **g.-649 A>G-R** | **TGTGATTTGGGCTATCCTTTACATTAAACTGTTCG** |  |
| **g.-491 C>A-F** | **CGCTAAAACCCCAGTAATGAAATATTTTGCCTTAATTGG** | **pGL3-*CgScd*:**  **-496C>A Single Site Mutation** |
| **g.-491 C>A-R** | **TTACTGGGGTTTTAGCGGGACTCGATCATATT** |  |
| **g.-389 T>C-F** | **CGACGTTTTGAAAGGAGTGACCATCCCTTGCA** | **pGL3-*CgScd*:**  **-389T>C Single Site Mutation** |
| **g.-389 T>C-R** | **CTCCTTTCAAAACGTCGTTATAACCAATTAAGGCAAAATATTTCA** |  |
| **g.-354 G>C-F** | **GGAGTGACTATCCCTTGCAGAACTAGTCTTTACTG** | **pGL3-*CgScd*:**  **-354G>C Single Site Mutation** |
| **g.-354 G>C-R** | **CAAGGGATAGTCACTCCTTTCAAAACGTCCTTAT** |  |
| **g.-333 C>T-F** | **GCAGAACTAGTCTTTGCTGAAATAAATGATCGATGTTGATACA** | **pGL3-*CgScd*:**  **-333C>T Single Site Mutation** |
| **g.-333 C>T-R** | **GCAAAGACTAGTTCTGCAAGGGATGGTCACTC** |  |
| **g.-310 A>G-F** | **CAGAAATATTAGAGAGAAGCTTATATACTGTATACAGGAAAA** | **pGL3-*CgScd*:**  **-310A>G Single Site Mutation** |
| **g.-310 A>G-R** | **TCTCTCTAATATTTCTGTATCAACATCGATCATTTATT** |  |
| **g.-275 G>T-F** | **AAATATGAAAGAGAAGCTTATATACTGTATACAGGAAAATATT** | **pGL3-*CgScd*:**  **-275G>T Single Site Mutation** |
| **g.-275 G>T-R** | **GCTTCTCTTTCATATTTCTGTATCAACATCGATCATT** |  |
| **g.-273 G>A-F** | **GAGAAGCTTATATAATGTATACAGGAAAATATTTGCCCCT** | **pGL3-*CgScd*:**  **-273G>A**  **Single Site Mutation** |
| **g.-273 G>A-R** | **CATTATATAAGCTTCTCTCTCATATTTCTGTATCA** |  |
| **g.-257 C>A-F** | **CTTTTTGTCAGTAGGCGAATTTAAAACTTGGCAA** | **pGL3-*CgScd*:**  **-257C>A Single Site Mutation** |
| **g.-257 C>A-R** | **CGCCTACTGACAAAAAGGGCGAAAGGGGTGAA** |  |
| **g.-198 C>T-F** | **CAAGGGTCTGTACCCCAGGAGGTGGAGGACTC** | **pGL3-*CgScd*:**  **-198C>T Single Site Mutation** |
| **g.-198 C>T-R** | **TGGGGTACAGACCCTTGGGCGGGCACTGGCTC** |  |
| **pET28a-*Cg*YB-F** | **GAGCTCACGGGGACGGTGAAGTGGTT** | ***Cg*YB protein purification** |
| **pET28a-*Cg*YB-R** | **CTCGAGAGGTCCGGTGACATTGG** |  |
| **pET-28a-*Cg*SREBP-F** | **GAGCTCATGGAGAACGTGGAAGG** | ***Cg*SREBP protein purification** |
| **pET-28a-*Cg*SREBP-F** | **CTCGAGCCTAGTTTGATCTCT** |  |

Supplementary Table 3 Primer sequences for plasmid construction, high-throughput sequencing and Sanger sequencing. The lowercase represents the sequences of the plasmid.

| **Name** | **Probe sequence(5′-3′)** |
| --- | --- |
| **g. -333 C -F** | **Biotin-**GACGTTTTGAAAGGAGTGACCATCCCTTGCAGAACTAGTCT |
| **g. -333 C -R** | AGACTAGTTCTGCAAGGGATGGTCACTCCTTTCAAAACGTC |
| **g. -333 T -F** | **Biotin-**GACGTTTTGAAAGGAGTGACTATCCCTTGCAGAACTAGTCT |
| **g. -333 T -R** | AGACTAGTTCTGCAAGGGATAGTCACTCCTTTCAAAACGTC |
| **g. -273 C -F** | **Biotin-**ATGTTGATACAGAAATATGAGAGAGAAGCTTATATACTGTA |
| **g. -273 C -R** | TACAGTATATAAGCTTCTCTCTCATATTTCTGTATCAACAT |
| **g. -273 T -F** | **Biotin-**ATGTTGATACAGAAATATGAAAGAGAAGCTTATATACTGTA |
| **g. -273 T -R** | TACAGTATATAAGCTTCTCTTTCATATTTCTGTATCAACAT |
| **g. -257 C -F** | **Biotin-**ATGAGAGAGAAGCTTATATACTGTATACAGGAAAATATTTG |
| **g. -257 C -R** | CAAATATTTTCCTGTATACAGTATATAAGCTTCTCTCTCAT |
| **g. -257 T -F** | **Biotin-**ATGAGAGAGAAGCTTATATAATGTATACAGGAAAATATTTG |
| **g. -257 T -R** | CAAATATTTTCCTGTATACATTATATAAGCTTCTCTCTCAT |
| **g. -198 C -F** | **Biotin-**CCTTTCGCCCTTTTTGTCAGCAGGCGAATTTAAAACTTGGC |
| **g. -198 C -R** | GCCAAGTTTTAAATTCGCCTGCTGACAAAAAGGGCGAAAGG |
| **g. -198 T -F** | **Biotin-**CCTTTCGCCCTTTTTGTCAGTAGGCGAATTTAAAACTTGGC |
| **g. -198 T -R** | GCCAAGTTTTAAATTCGCCTACTGACAAAAAGGGCGAAAGG |

Supplementary Table 4 Probe sequences for DNA Pull-Down. The “biotin” represents the probes are labeled with biotin at 5’ end.

|  | **C16:1 desaturation index %** | **C18:1 desaturation index %** |
| --- | --- | --- |
| **Control** | 9.859±0.4330 | 50.390±0.7238 |
| **RNAi** | 7.960±0.2649 ** | 45.711±1.2989 * |

Supplementary Table 5 The palmitoleic acid (C16:1) and oleic acid (C18:1) desaturation indexes in RNAi experiments. Significant differences between groups were marked with * *p* < 0.05, ** *p* < 0.01, and *** *p* < 0.001.

|  | **C16:1 desaturation index %** | **C18:1 desaturation index %** |
| --- | --- | --- |
| **Control** | 19.919±0.5596 | 28.137±0.4599 |
| **pYES3-*CgScd*** | 22.980±0.2952 ** | 32.489±0.8407 ** |

Supplementary Table 6 The palmitoleic acid (C16:1) and oleic acid (C18:1) desaturation indexes in overexpression experiments. Significant differences between groups were marked with * *p* < 0.05, ** *p* < 0.01, and *** *p* < 0.001.

|  | **C16:1 desaturation index %** | **C18:1 desaturation index %** |
| --- | --- | --- |
| **Control** | 14.469±0.3488 | 78.816±0.7328 |
| **pcDNA3.1-*CgScd*** | 15.491±0.3041 * | 80.649±0.5682 * |

Supplementary Table 7 The palmitoleic acid (C16:1) and oleic acid (C18:1) desaturation indexes in fluorescence recovery after photobleaching (FRAP) experiments. Significant differences between groups were marked with * *p* < 0.05, ** *p* < 0.01, and *** *p* < 0.001.

| **Sample** | **Clean Reads** | **Clean Base(G)** | **Q30(%)** | **GC Content(%)** | **Average**  **Depth** | **Covered Ratio(%)** |
| --- | --- | --- | --- | --- | --- | --- |
| **Ca** | 4236451 | 1.27 | 91.78 | 35.44 | 1532X | 100% |
| **Cg** | 4236451 | 1.26 | 92.53 | 35.65 | 217X | 100% |

Supplementary Table 8 Statistics of the mixed pool target amplicon sequencing data.

| **C16:1 desaturation index %** | **Control** | **4℃ Treatment** |
| --- | --- | --- |
| ***C. gigas*** | 9.834±0.779 | 10.986±0.742 |
| ***C. angulata*** | 6.05914±2.017 | 6.899±2.051 |
| **C18:1 desaturation index %** | **Control** | **4℃ Treatment** |
| ***C. gigas*** | 8.886±0.060 | 9.674±0.309 |
| ***C. angulata*** | 8.198±0.111 | 9.315±0.403 |

Supplementary Table 10 The palmitoleic acid (C16:1) and oleic acid (C18:1) desaturation indexes in *C. gigas* and *C. angulata* during short-term cold stress.

**Reference**

Erdős G, Pajkos M, Dosztányi Z. 2021. IUPred3: prediction of protein disorder enhanced with unambiguous experimental annotation and visualization of evolutionary conservation. Nucleic Acids Research 49:W297-W303.

Letunic I, Khedkar S, Bork P. 2021. SMART: recent updates, new developments and status in 2020. Nucleic Acids Research 49:D458-D460.
